# A Multiple Imputation Approach to Distinguish Curative From Life‐Prolonging Effects in the Presence of Missing Covariates

**DOI:** 10.1002/bimj.70144

**Published:** 2026-06-08

**Authors:** Marta Cipriani, Marta Fiocco, Marco Alfò, Maria Quelhas, Eni Musta

**Affiliations:** ^1^ Department of Statistical Sciences Sapienza University of Rome Rome Italy; ^2^ Leiden University, Mathematical Institute Leiden The Netherlands; ^3^ Department of Biomedical Data Science Leiden University Medical Centre Leiden The Netherlands; ^4^ Princess Maxima Centre for Pediatric Oncology, Trial and Data Center Utrecht The Netherlands; ^5^ University of Amsterdam, Korteweg‐de Vries Institute for Mathematics Amsterdam The Netherlands

**Keywords:** chained equations, mixture cure models, multiple imputation, osteosarcoma

## Abstract

Medical advances have increased cancer survival rates and the possibility of finding a cure. Hence, it is crucial to evaluate the impact of treatments both in terms of cure and prolongation of survival. To achieve this, we may use a Cox proportional hazards cure model. However, a significant challenge in applying such a model is the potential presence of partially observed covariates. We aim to refine the methods for imputing partially observed covariates based on multiple imputation and fully conditional specification approaches. To be more specific, we consider a general case in which different covariate vectors are used to model the probability of cure and the survival of patients who are not cured. In a large‐scale simulation experiment, we investigated the performance of the multiple imputation procedure based either on the exact conditional distribution or on an approximate imputation model, which helps to draw imputed values at a lower computational cost. To assess the effectiveness of these approaches, we compare them with a complete‐case analysis and an analysis that includes all available covariates in modeling both cure probabilities and the survival of the uncured. We discuss the application of these techniques to a real‐world dataset from the BO06 clinical trial on osteosarcoma.

## 1 Introduction

Recent advances in medicine have increased the chances of survival for some types of cancer, such as breast cancer, melanoma, osteosarcoma, and acute childhood lymphocytic leukemia, and have even made cure possible. As a result, it is important not only to estimate the impact that several risk factors may have on survival, but also to analyze their effect on the probability of being cured. This is especially relevant in pediatric cancer. Osteosarcoma, a type of bone tumor that primarily affects children and young adults, may help illustrate this need. For this type of cancer, more than 95% of patients who do not experience a relapse within 5 years after treatment are considered cured and are unlikely to relapse later (Ferrari et al. 2006). In survival analysis, individuals who will never experience the event of interest, such as disease recurrence or disease‐related death, are referred to as “cured.” Hence, it is crucial to consider cured patients in statistical analyses to assess the efficacy of a treatment in terms of cure rather than only focus on its impact on prolonging survival.

Traditional survival analysis methods can be less informative when a portion of the population is considered cured, as they may not account for differences in cure status within the population. Furthermore, the cure status is not always observable; while we know that patients who experience the event of interest are uncured, we cannot determine whether censored patients will eventually experience the event. This led to the development of cure models, which provide a more suitable modeling approach, particularly in oncological studies (Legrand and Bertrand 2019).

One type of cure model is the mixture cure model, which defines overall survival as a combination of the probability of cure (incidence) and the survival of uncured patients (latency). Mixture cure models allow for the use of different covariates when modeling the incidence and latency components. This means that we can differentiate between the effect of a covariate on the probability of being cured and its effect on the survival of uncured patients. In this context, we consider a Cox proportional hazards (PH) cure model, where the incidence is modeled by a logistic regression model and a Cox PH model is used for the latency. Estimation is generally performed using the maximum likelihood approach in an incomplete data setting. For this purpose, the expectation‐maximization (EM) algorithm is often used due to the latent cure status (Cai et al. 2012). For more detailed information on cure models, see the review paper by Amico and Van Keilegom (2018) or the book by Peng and Yu (2021).

A recent study by Musta et al. (2022) examined the prognostic importance of the histological response and intensified chemotherapy on the cure rate and progression‐free survival (PFS) in a sample of osteosarcoma patients who have not been cured. However, the study excluded observations with missing histological responses from the analysis. This motivated us to investigate, in this article, the sensitivity of the results to the method used to handle missing values in the histological response.

When working with an incomplete dataset, a straightforward approach is to perform a complete‐case analysis (CCA), which involves removing observations with missing data. However, this method can lead to inefficient results and, depending on the missing data mechanism and the model of interest, potentially biased estimates (Little and Rubin 2002). In particular, while CCA is guaranteed to be unbiased under missing completely at random (MCAR), it can also yield valid results under missing at random (MAR) in certain model settings, such as when the missingness mechanism does not depend on the outcome when conditioning on the covariates (Bartlett et al. 2015; Hughes et al. 2019). Alternatively, missing values can be filled in by using simple imputation techniques, either based on summaries (e.g., mean) or statistical models (e.g., regression) based on available data. While these methods allow the use of the entire sample, completed by imputation, they may underestimate the standard errors of the final model (van Buuren 2018). To address these issues, Rubin (1987) introduced multiple imputation techniques. Multiple imputation involves creating multiple imputed datasets, estimating the parameters in each of them separately, and then combining the results. This approach produces unbiased estimates and incorporates the uncertainty of imputation into standard error estimates, provided that the missing data mechanism satisfies the MAR assumption. However, it should be noted that in settings where data are missing not at random (MNAR), both CCA and multiple imputation can produce biased results (Bartlett et al. 2015), and in some cases CCA may be preferable to multiple imputation (Hughes et al. 2019). For guidance on assessing the validity of these approaches, see general guidelines on the treatment and reporting of missing data (Lee et al. 2021).

When performing multiple imputation, it is generally recommended to include in the imputation model the outcome of the analysis model, as well as all other variables of the analysis model. When handling survival data in particular, a suitable representation of the survival outcome should be used (White and Royston 2009). In the case of Cox PH models, White and Royston (2009) advocate a particular representation of the outcome to yield approximately compatible imputation models, and, more generally, the substantive model–compatible approach provides a framework to perform multiple imputation that respects the structure of Cox PH models (Bartlett et al. 2015).

However, dealing with cured patients presents an additional challenge for the imputation procedure. Beesley et al. (2016) have developed a method for using multiple imputation in the Cox PH cure model, which is based on specifying either an exact or an approximate distribution of the missing covariates, conditional on the available ones and the survival response (time, status). The authors state in that paper that, for simplicity, they assume the same design vector is used in the models for both incidence and latency. This assumption restricts the applicability of the method in practice, as including all available covariates in both components of the mixture model may lead to the estimation of a large number of parameters. In addition, the factors affecting the probability of being cured can differ from those influencing the hazard, making the incidence and latency settings distinct from each other. Moreover, as demonstrated by Hanin and Huang (2014), the sharing of covariates between various components of a Cox PH cure model may prevent the model identifiability. Hence, a simpler model could be preferred (over the one with all covariates in both components) whenever supported by the data.

In this paper, we consider the more general case of a mixture cure model with possibly different sets of covariates for the incidence and the latency component. This allows us to fully leverage the benefits of a mixture model. Specifically, we consider three cases depending on whether the variable with missing values affects only the probability of cure, only the survival of the uncured, or both components. Building on the work of Beesley et al. (2016), we derive approximate imputation models for these scenarios. In particular, only for the latter one, the approximate model is the same as in Beesley et al. (2016). Through simulation studies, we investigate how these two multiple imputation approaches perform in practice in this more general setting, an aspect not explored in the original work by Beesley et al. (2016). The simulation results also emphasize the need to differentiate between the covariates modeling the probability of cure and the survival of the uncured patients, as including all covariates in both components leads to wider confidence intervals (CIs). However, model selection is not a trivial task in the presence of missing values and model misspecification seems to deteriorate the performance of the exact imputation method. However, the approximate imputation model appears to be quite robust to model misspecification and would be the recommended choice despite the slightly wider CIs compared to the exact approach. Lastly, to facilitate the application of our method in practice, we provide general‐purpose software.^1^

In Section 2, we introduce the fundamental concepts underlying cure models and the use of multiple imputation in this context. In Section 3, we outline the methodology for performing multiple imputation in cure models when the sets of covariates for incidence and latency models are different. In Section 4, we evaluate the performance of this method using simulated data. In Section 5, we apply the developed methodology to analyze osteosarcoma data from the BO06 clinical trial and compare the results with the complete‐case approach. Finally, in Section 6, we conclude with a discussion.

## 2 Basic Concepts

### 2.1 Mixture Cure Models

We are interested in studying the time T until an event of interest occurs, in a sample consisting of cured and uncured individuals. The cured individuals will not experience the event of interest during their lifetime. We denote by G the binary indicator of the uncured status, that is, G=1 if uncured and G=0 if cured. However, due to the censoring at time C and to the limited time window covered by the study at hand, these variables cannot be directly observed. Instead, we observe the follow‐up time Y=min(T,C) and the event indicator Δ=I(T<C). For uncensored observations (Δ=1), G=1, while for censored observations G is unknown.

We consider a mixture cure model, which expresses overall survival as a combination of the probability of being cured (with potentially infinite survival) and the survival of the uncured individuals. The probability of being uncured, also known as incidence, given a design vector $X\in R^{p}$, is denoted by π(X)=P(G=1|X), and is typically described by a logistic regression function (Farewell 1977; Legrand and Bertrand 2019)

(1) $$\pi \left(X\right)=\frac{\exp\left(\alpha_{0}+\alpha^{T}X\right)}{1+\exp\left(\alpha_{0}+\alpha^{T}X\right)},$$

where $\alpha_{0}\in R$ is the intercept and $\alpha \in R^{p}$ denotes the vector of regression parameters.

The survival of the uncured patients, known as latency, conditional on a set of covariates $Z\in R^{q}$, which may differ from X, is represented by $S_{u}\left(t|Z\right)=P\left(T>t|Z,G=1\right)$, and it can be modeled employing a regression model such as the Cox PH model (Cox 1972)

(2) $$S_{u}\left(t|Z\right)=\exp\left(-H_{0}\left(t\right)\exp\left(\beta^{T}Z\right)\right),$$

where $H_{0}\left(\cdot \right)$ denotes the cumulative baseline hazard function of the uncured and $\beta \in R^{q}$ is the vector of regression parameters. In this model, the PH assumption holds for the subpopulation of uncured patients, but not for the entire population. Combining these components (Kuk and Chen 1992), the overall survival is given by

(3) $$S\left(t|X,Z\right)=\left(1-\pi \left(X\right)\right)+\pi \left(X\right)S_{u}\left(t|Z\right).$$

This model is referred to as the Cox PH cure model.

For a cure model to be identifiable, a sufficiently long follow‐up is required, that is, the study duration should be longer than the support of the distribution for the event times of the uncured individuals. For a more extensive discussion of the assumption of sufficient follow‐up, we refer the reader to the review by Amico and Van Keilegom (2018). In practice, to check whether such an assumption is satisfied, one typically combines medical knowledge with a visual inspection of the Kaplan–Meier estimate. Ideally, we would like to observe a long plateau after the last observed event, which contains many censored observations. Recently, some statistical tests have also been proposed for the sufficient follow‐up assumption (Maller et al. 2024; Xie et al. 2023; Yuen and Musta 2024).

The complete‐data likelihood function for the Cox PH cure model can be written as

(4) $$\begin{array}{ccc} L_{c}\left(\alpha_{0},\alpha ,\beta ,H_{0}\right) & = & \prod_{i=1}^{n}{\left[\pi \left(X_{i}\right)f_{u}\left(Y_{i}|Z_{i}\right)\right]}^{\Delta_{i}G_{i}}\\ & & \times \;{\left[\pi \left(X_{i}\right)S_{u}\left(Y_{i}|Z_{i}\right)\right]}^{\left(1-\Delta_{i}\right)G_{i}}{\left[1-\pi (X_{i})\right]}^{\left(1-\Delta_{i}\right)\left(1-G_{i}\right)}, \end{array}$$

where $f_{u}\left(\cdot \right)$ denotes the probability density function of the uncured population. This likelihood can be factorized into two components, containing the parameters of the incidence and the latency, respectively.

Since this model depends on the latent cure status G, the set of parameters is usually estimated using an EM algorithm (Peng and Dear 2000; Sy and Taylor 2000), which is an iterative two‐step procedure. In the E‐step, the posterior expectation of the complete data log‐likelihood over the latent uncured indicator is obtained, conditional on the parameters estimated from the previous iteration. For individual i∈(1,…n) at iteration r, this is given by

$$\begin{array}{cc} q_{i}^{\left(r\right)} & =E(G_{i}|Y_{i},\Delta_{i},X_{i},Z_{i},\alpha_{0}^{\left(r-1\right)},\alpha^{\left(r-1\right)},\beta^{\left(r-1\right)},H_{0}^{\left(r-1\right)})\\ & =\Delta_{i}+\left(1-\Delta_{i}\right)\frac{\pi^{\left(r-1\right)}\left(X_{i}\right)S_{u}^{\left(r-1\right)}\left(Y_{i}|Z_{i}\right)}{1-\pi^{\left(r-1\right)}\left(X_{i}\right)+\pi^{\left(r-1\right)}\left(X_{i}\right)S_{u}^{\left(r-1\right)}\left(Y_{i}|Z_{i}\right)}, \end{array}$$

where $\pi^{\left(r-1\right)}$ and $S_{u}^{\left(r-1\right)}$ are computed according to Equations (1) and (2) by using the parameter estimates resulting from iteration r−1, namely, $\alpha_{0}^{\left(r-1\right)},\alpha^{\left(r-1\right)},\beta^{\left(r-1\right)},H_{0}{\left(\cdot \right)}^{\left(r-1\right)}$. The M‐step of the EM algorithm consists of maximizing the expected log‐likelihood for complete data given in (4), where, at each iteration, $G_{i}$ is replaced by its expectation $q_{i}^{\left(r\right)}$. This reduces to a simpler weighted data problem, with an expected log‐likelihood which can be maximized by standard algorithms.

If parameter distinctiveness holds, the two components of the expected likelihood can be maximized separately, one with respect to $\alpha_{0}$ and α, and the other with respect to β and $H_{0}\left(\cdot \right)$. Maximization with respect to $H_{0}\left(\cdot \right)$ at iteration r leads to the following modified Breslow estimator (Breslow 1974) of the baseline cumulative hazard function

(5) $$\begin{array}{c} {\hat{H}}_{0}^{\left(r\right)}\left(t\right)=\sum_{t_{j}\leq t}\frac{D(t_{j})}{\sum_{i\in R_{j}}q_{i}^{\left(r\right)}\exp\left(\beta^{(r-1)T}Z_{i}\right)}, \end{array}$$

where $D(t_{j})$ is the number of events at time $t_{j}$ and $R_{j}$ represents the set of observations at risk at time $t_{j}$ (Sy and Taylor 2000).

### 2.2 Handling Missing Values by Multiple Imputation

We first illustrate the multiple imputation approach for a general model with outcome Y and design vector $X\in R^{p}$, where $X^{\left(j\right)}$ has missing observations. The procedure consists of imputing the incomplete covariate $X^{\left(j\right)}$ by defining a model for this element, conditional on the outcome Y and the remaining covariates $X^{\left(-j\right)}=X\setminus X^{\left(j\right)}$. This is done using the fully conditional specification (FCS) approach (van Buuren et al. 2006), which requires the specification of the conditional distribution $f(X^{\left(j\right)}|Y,X^{\left(-j\right)};\theta$), where θ represents a set of parameters. Such parameters depend on j, but we will omit it from the notation for simplicity. The procedure begins by fitting a regression model for $X^{\left(j\right)}$ with covariates Y and $X^{\left(-j\right)}$ on the portion of observed data for $X^{\left(j\right)}$. This provides us with estimates $\hat{\theta }$ for the regression coefficients $\theta \in R^{p}$, as well as the estimated covariance matrix ${\hat{\Sigma }}_{\theta }$. We then draw values $\theta^{*}$ from the posterior distribution of θ given the observations, which is approximated by a Gaussian distribution, $N(\hat{\theta },{\hat{\Sigma }}_{\theta })$. The imputed values for the cases where $X^{\left(j\right)}$ is missing are drawn from the conditional distribution $f(X^{\left(j\right)}|Y,X^{\left(-j\right)};{\hat{\theta }}^{*})$. This step is repeated K times, resulting in K imputed samples.

**FIGURE 1:**
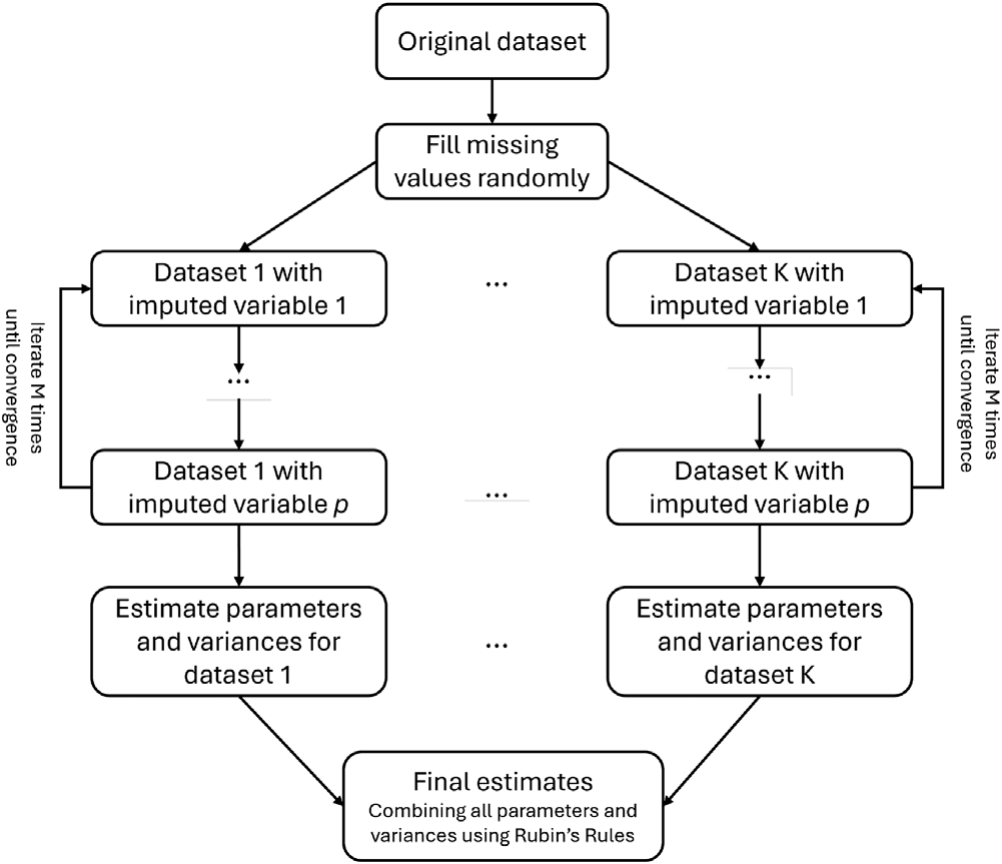
Multiple imputation by chained equations procedure.

When there are multiple variables with missing observations, this process is repeated sequentially for each one of them, using the observed and imputed values of the remaining variables. This procedure, known as multiple imputation by chained equations, is summarized in Figure 1. It starts by filling all missing values randomly and then creates K datasets with imputed values for incomplete variables. The order in which the variables are imputed is determined by the proportion of missing cases, starting with the variable that has the least missing data (van Buuren 2018). Each variable uses the observed and most recently imputed values of the remaining ones, and this method is iterated on each dataset until convergence.

After obtaining K imputed datasets, the set of parameters of interest Ψ is estimated separately on each of these datasets, resulting in a vector of estimates $\left({\hat{\Psi }}_{1},\text{…},{\hat{\Psi }}_{K}\right)$ and estimated covariance matrices $\left({\hat{\Sigma }}_{\Psi 1},\text{…},{\hat{\Sigma }}_{\Psi K}\right)$. Finally, the pooled estimate is obtained by combining the K estimates using Rubin's rule (Rubin 2004)

$$\begin{array}{c} \hat{\Psi }=\frac{1}{K}\sum_{k=1}^{K}\Psi_{k} \end{array}$$

and the final covariance matrix is given by

$$\begin{array}{cc} {\hat{\Sigma }}_{\Psi } & =\frac{1}{K}\sum_{k=1}^{K}\Sigma_{\Psi k}+\left(1+\frac{1}{K}\right)\frac{1}{K-1}\sum_{k=1}^{K}\left(\Psi_{k}-\hat{\Psi }\right){\left(\Psi_{k}-\hat{\Psi }\right)}^{T}, \end{array}$$

where the first component is the average of the repeated complete‐data posterior covariances of Ψ (within component), and the second component is the estimate of the covariance between the K complete‐data posterior means of Ψ (between component).

### 2.3 Multiple Imputation for Cure Models

To perform multiple imputation in the case of a cure model with missing covariates, the first step is to determine the conditional distribution of the covariate with missing values given the outcome and the other covariates. For survival data, the outcome consists of both the follow‐up time Y and the event indicator Δ. In the case of the standard Cox model (without cure fraction), it has been shown that a suitable model for imputing the missing covariate values is based on Δ, the cumulative baseline hazard $H_{0}\left(Y\right)$, and the other covariates (White and Royston 2009).

However, for the cure model, an additional challenge arises from the fact that even the cure status G is unobserved for censored observations. For a Cox PH cure model where the design vector is common to both the incidence and the latency (i.e., X=Z), Beesley et al. (2016) derived the exact conditional distribution based on the complete likelihood function in (4). They proposed a chained equations procedure which imputes iteratively the missing values for the cure status G and the incomplete covariates. In practice, drawing observations from the exact conditional distribution, which has a complicated form, is often computationally expensive. To reduce the computational time, Beesley et al. (2016) proposed approximations of the exact conditional distribution which are easier to handle in practice. However, assuming that X and Z are the same is quite restrictive, preventing us from fully exploiting the advantage of a mixture cure model in distinguishing between covariates with a curative or just a life‐prolonging effect.

The ideas of Beesley et al. (2016) have been explicitly discussed in this limited context, but we think they can be extended to the more general model described in Section 2.1, considering three possible scenarios: the incomplete covariate belongs only to X, only to Z, or to both X and Z. The scenario where the variable with missing values belongs to both X and Z is treated in Section 3, while the cases where it belongs to only one of the two components (either the incidence or the latency one) are discussed in the appendices. Specifically, Appendix A deals with the case using the exact conditional distribution, while Appendix B addresses the case of the approximate conditional distribution. Further details about the method proposed by Beesley et al. (2016) and how it relates to this work are given in Section 3.

## 3 Methodology

In this section, we describe the multiple imputation procedure in cure models with incomplete covariates. As mentioned in Section 2.2, the main aspect of the multiple imputation procedure involves generating imputed values based on the distribution of the incomplete variable conditional on the other ones. Therefore, we first present the exact form of the conditional distribution and then outline the steps of the algorithm. Finally, we introduce an approximation of the conditional distribution that enables us to generate imputed values at a reduced computational cost.

### 3.1 Using the Exact Conditional Distribution

Let W∈R be the incomplete variable present in both the incidence and the latency model. The results for the cases where W belongs either to the incidence or to the latency model are discussed in the Appendix. Since W belongs to both X and Z, there exist 1≤j≤p and 1≤l≤d such that $W=X^{\left(j\right)}=Z^{\left(l\right)}$. We denote the set of incidence and latency covariates excluding W as $X^{\left(-j\right)}=X\setminus X^{\left(j\right)}$ and $Z^{\left(-l\right)}=Z\setminus Z^{\left(l\right)}$, respectively. To detail the proposed procedure in a more general context, we will consider two different choices for W, either having a Gaussian or a Bernoulli distribution, conditional on the other covariates. However, results can be similarly generalized to other distributions.

We note also that the following formulas can be obtained from Equation (2) in Beesley et al. (2016) by including all covariates to both parts of the model with corresponding coefficients equal to zero for the nonactive covariates. However, although this reparameterization is mathematically equivalent, it is not equivalent from an estimation perspective. Including an *inactive* covariate in a model component (even with a true coefficient equal to zero) requires estimating an additional parameter within the EM algorithm and alters both the dimensionality of the optimization step and the structure of the information matrix. This leads to slower convergence and increased numerical instability compared with fitting the model only with the covariates that are truly involved in each component. For this reason, we implement the model by including each covariate only in the part of the cure model where it is assumed to have an effect.

*Case 1*. Let us first assume that $W|X^{\left(-j\right)},Z^{\left(-l\right)}$ follows a normal distribution, and that, at least approximately, the dependence of W on $X^{\left(-j\right)}$ and $Z^{\left(-l\right)}$ is summarized by a change in the location (mean) parameter only. That is, we specify:

$$\begin{array}{c} W|X^{\left(-j\right)},Z^{\left(-l\right)}∼N\left(\mu ,\sigma \right), \end{array}$$

where

(6) $$\begin{array}{c} \mu =\theta_{0}+\sum_{b\neq j}^{p}\theta_{b}X_{b}+\sum_{\begin{array}{c} s\neq l\\ Z_{s}\notin X \end{array}}^{d}\theta_{p+s}Z_{s}. \end{array}$$

Note that we only consider variables in Z that are not in X ($Z_{s}\notin X$) to prevent any useless overlapping information. In this case, the complete data likelihood is given by

(7) $$\begin{array}{cc} & L(\alpha_{0},\alpha ,\beta ,H_{0},\mu ,\sigma )\\ & \;=\prod_{i=1}^{n}{\left[\pi \left(X_{i}\right)f_{u}\left(Y_{i}|Z_{i}\right)\right]}^{\Delta_{i}G_{i}}{\left[\pi \left(X_{i}\right)S_{u}\left(Y_{i}|Z_{i}\right)\right]}^{\left(1-\Delta_{i}\right)G_{i}}\\ & \;\;\times {\left[1-\pi (X_{i})\right]}^{\left(1-\Delta_{i}\right)\left(1-G_{i}\right)}f\left(W_{i}|X_{i}^{\left(-j\right)},Z_{i}^{\left(-l\right)}\right)\\ & \;\;=\prod_{i=1}^{n}{\left[\frac{e^{\alpha_{0}+\alpha^{T}X_{i}}}{1+e^{\alpha_{0}+\alpha^{T}X_{i}}}{\left(h_{0}\left(Y_{i}\right)e^{\beta^{T}Z_{i}}\right)}^{\Delta_{i}}e^{-H_{0}\left(Y_{i}\right)e^{\beta^{T}Z_{i}}}\right]}^{G_{i}}\\ & \;\;\times {\left(\frac{1}{1+e^{\alpha_{0}+\alpha^{T}X_{i}}}\right)}^{1-G_{i}}\left(e^{\frac{-{\left(W_{i}-\mu_{i}\right)}^{2}q}{2\sigma^{2}}}\right). \end{array}$$

Therefore, the exact distribution of W conditional on all the remaining covariates is as follows:

(8) $$\begin{array}{cc} f(W_{i}|X_{i}^{\left(-j\right)},Z_{i}^{\left(-l\right)},Y_{i},\Delta_{i},G_{i}) & \propto {\left(\frac{e^{\alpha_{0}+\alpha^{T}X_{i}}}{1+e^{\alpha_{0}+\alpha^{T}X_{i}}}e^{\beta^{T}Z_{i}\Delta_{i}}e^{-H_{0}\left(Y_{i}\right)e^{\beta^{T}Z_{i}}}\right)}^{G_{i}}\\ & \times {\left(\frac{1}{1+e^{\alpha_{0}+\alpha^{T}X_{i}}}\right)}^{1-G_{i}}\left(e^{\frac{-{\left(W_{i}-\mu_{i}\right)}^{2}}{2\sigma^{2}}}\right). \end{array}$$

One can draw values to impute W from this distribution using a Metropolis–Hastings algorithm (Hastings 1970; Metropolis et al. 1953). This algorithm can draw values from a given generic probability distribution (the target distribution) by sampling from a Markov chain whose stationary distribution is the target distribution.

*Case 2*. Alternatively, $W|X^{\left(-j\right)},Z^{\left(-l\right)}$ could be a categorical random variable. For instance, if W is binary, we can specify:

$$\begin{array}{c} W|X^{\left(-j\right)},Z^{\left(-l\right)}∼\text{Ber}\left(\mathrm{expit}\left(\mu \right)\right), \end{array}$$

where $\mathrm{expit}\left(x\right)=e^{x}/\left(1+e^{x}\right)$ and μ is given by (6). The complete data likelihood is as in (7) where $f(W_{i}|X_{i}^{\left(-j\right)},Z_{i}^{\left(-l\right)})$ is replaced by $P(W_{i}=w_{i}|X_{i}^{\left(-j\right)},Z_{i}^{\left(-l\right)})$ with $w_{i}\in \left\{0,1\right\}$. Recalling that $\mathrm{logit}\left(x\right)=\log\left(\frac{x}{1-x}\right)$, the exact distribution of W conditional on all the remaining covariates takes the form

(9) $$\begin{array}{cc} & \mathrm{logit}\left[P(W_{i}=1|X_{i}^{\left(-j\right)},Z_{i}^{\left(-l\right)},Y_{i},\Delta_{i},G_{i})\right]\\ & \;=\log\left[\frac{P(W_{i}=1|X_{i}^{\left(-j\right)},Z_{i}^{\left(-l\right)},Y_{i},\Delta_{i},G_{i})}{P(W_{i}=0|X_{i}^{\left(-j\right)},Z_{i}^{\left(-l\right)},Y_{i},\Delta_{i},G_{i})}\right]\\ & \;=G_{i}\Delta_{i}\beta_{l}-G_{i}H_{0}\left(Y_{i}\right)e^{\sum_{s\neq l}^{d}\beta_{s}Z_{i,s}}\left(e^{\beta_{l}}-1\right)+G_{i}\alpha_{j}+\\ & \;+\log\left(1+e^{\alpha_{0}+\sum_{b\neq j}^{p}\alpha_{b}X_{i,b}}\right)+\\ & \;-\log\left(1+e^{\alpha_{0}+\alpha_{j}+\sum_{b\neq j}^{p}\alpha_{b}X_{i,b}}\right)+\mu_{i}. \end{array}$$

Given that the conditional distributions depend on μ in both cases, one must draw values $\theta^{*}$ for the parameters θ before drawing values for W. This is done by running a regression model (logistic regression when $W|X^{\left(-j\right)},Z^{\left(-l\right)}∼$ Bernoulli and linear regression when $W|X^{\left(-j\right)},Z^{\left(-l\right)}∼$ Normal), obtaining point estimates $\hat{\theta }$ and the corresponding covariance matrix estimates ${\hat{\Sigma }}_{\theta }$. Then, we draw values $\theta^{*}$ from a Normal $\left(\hat{\theta },{\hat{\Sigma }}_{\theta }\right)$ distribution.

We note that the conditional models specified above (Normal or Bernoulli) should be interpreted as working models for the distribution of $W|X^{\left(-j\right)},Z^{\left(-l\right)}$. This reflects the practical setting in which the true conditional distribution is typically unknown. As discussed later in the simulation study, even when the marginal distribution of W is generated from a known form (Normal or Bernoulli), the resulting conditional distribution remains an approximation of the true data‐generating mechanism.

### 3.2 Implementing Multiple Imputation in Cure Models

Let us consider the Cox PH cure model described in Section 2.1. To impute missing observations for any incomplete covariate by drawing values from its conditional distribution, we need to know G and all the model parameters. Therefore, we use an iterative procedure where we first estimate the parameters, impute G, and then impute the missing covariate values. The pseudo‐code of the corresponding algorithm is provided in Algorithm 1, and it can be briefly described as follows:

ALGORITHM 1 **Imputation of missing covariates using exact conditional distribution**

**Table:** 

**Require:** dataset of n>0 observations; number of iterations for chained equations M>0; number of imputed data sets K>0; $W|X^{\left(-j\right)},Z^{\left(-l\right)}∼N\left(\mu ,\sigma \right)$ or $W|X^{\left(-j\right)},Z^{\left(-l\right)}∼Ber\left(\mathrm{expit}\left(\mu \right)\right)$
**Ensure:** Imputed data for W
**0:** **Initialization** (m=0)
Assign values to $\alpha_{0}^{\left(0\right)},\alpha^{\left(0\right)},\beta^{\left(0\right)},H_{0}^{\left(0\right)}\left(t\right)$, and randomly fill the missing cases of W producing $W^{\left(0\right)}$
**for** m=1,⋯,M **do**
1: **Estimate** $H_{0}\left(t\right)$
**for** i=1,⋯,n **do**
**a:** Compute $\pi^{\left(m-1\right)}\left(X_{i}\right)=\frac{\exp(\alpha_{0}^{\left(m-1\right)}+\alpha_{W}^{\left(m-1\right)}W_{i}^{\left(m-1\right)}+\alpha_{X}^{(m-1)T}X_{i}^{\left(-j\right)})}{1+\exp(\alpha_{0}^{\left(m-1\right)}+\alpha_{W}^{\left(m-1\right)}W_{i}^{\left(m-1\right)}+\alpha_{X}^{(m-1)T}X_{i}^{\left(-j\right)})}$
**b:** Compute $S_{u}^{\left(m-1\right)}\left(t|Z_{i}\right)=\exp\left(-H_{0i}^{\left(m-1\right)}\left(t\right)\exp\left(\beta_{W}^{\left(m-1\right)}W_{i}^{\left(m-1\right)}+\beta_{Z}^{(m-1)T}Z_{i}^{\left(-l\right)}\right)\right)$
**c:** Compute $q_{i}^{\left(m\right)}=\Delta_{i}+\left(1-\Delta_{i}\right)\frac{\pi^{\left(m-1\right)}\left(X_{i}\right)S_{u}^{\left(m-1\right)}\left(t|Z_{i}\right)}{1-\pi^{\left(m-1\right)}\left(X_{i}\right)+\pi^{\left(m-1\right)}\left(X_{i}\right)S_{u}^{\left(m-1\right)}\left(t|Z_{i}\right)}$
**end for**
Get $H_{0}^{\left(m\right)}\left(Y_{i}\right)=\sum_{t_{j}<t_{i}}\frac{D(t_{j})}{\sum_{i\in R_{j}}q_{i}^{\left(m\right)}\exp\left(\beta_{W}^{\left(m-1\right)}W_{i}^{\left(m-1\right)}+\beta_{Z}^{(m-1)T}Z_{i}^{\left(-l\right)}\right)}$
2: **Draw values for** $\alpha_{0},\alpha ,\beta$
**a:** Compute $\hat{\alpha_{0}},\hat{\alpha }$ and ${\hat{\Sigma }}_{\left(\alpha_{0},\alpha \right)}$ from $\mathrm{logit}[P\left(G^{\left(m-1\right)}=1|W^{\left(m-1\right)},X^{\left(-j\right)}\right)]$
**b:** Draw $\left(\alpha_{0}^{\left(m\right)},\alpha^{\left(m\right)}\right)$ from MVN $\left(\left(\hat{\alpha_{0}},\hat{\alpha }\right),{\hat{\Sigma }}_{\left(\alpha_{0},\alpha \right)}\right)$
**c:** Compute $\hat{\beta }$ and ${\hat{\Sigma }}_{\beta }$ fitting $S_{u}\left(t|W^{\left(m-1\right)},Z^{\left(-l\right)}\right)$ to the subjects such that $G_{i}=1$
**d:** Draw $\beta^{\left(m\right)}$ from MVN $\left(\hat{\beta },{\hat{\Sigma }}_{\beta }\right)$
3: **Impute** G
**for all** $G_{i}=$ *NA* **do** draw from
$\mathrm{logit}[P\left(G_{i}=1|W_{i}^{\left(m-1\right)},X_{i}^{\left(-j\right)},Z_{i}^{\left(-l\right)},Y_{i},\Delta_{i}=0;\alpha_{0}^{\left(m\right)},\alpha^{\left(m\right)},\beta^{\left(m\right)}\right)]$
**end for**
4: **Impute W**
Specify $f(W_{i}|G_{i},\Delta_{i},Y_{i},X_{i}^{\left(-j\right)},Z_{i}^{\left(-l\right)};\theta )$ and
**if** W∼Ber(expit(μ)) **then**
**a:** Compute $\hat{\theta }$ and ${\hat{\Sigma }}_{\theta }$ from a logistic regression on $W_{i}^{\left(m-1\right)}$ using $X_{i}^{\left(-j\right)},Z_{i}^{\left(-l\right)}$
**b:** Draw values $\theta^{*}$ from MVN $\left(\hat{\theta },{\hat{\Sigma }}_{\theta }\right)$
**c:** **for all** $W_{i}^{\left(0\right)}=$ *NA* **do** draw imputed values from
$\mathrm{logit}[P\left(W_{i}=1|G_{i},\Delta_{i},Y_{i},X_{i}^{\left(-j\right)},Z_{i}^{\left(-l\right)};\theta^{*}\right)]$
**else**
**if** W∼N(μ,σ) **then**
**a:** Compute $\hat{\theta }$ and ${\hat{\Sigma }}_{\theta }$ from a linear regression on $W_{i}^{\left(m-1\right)}$ using $X_{i}^{\left(-j\right)},Z_{i}^{\left(-l\right)}$
**b:** Draw values $\theta^{*}$ from MVN $\left(\hat{\theta },{\hat{\Sigma }}_{\theta }\right)$
**c:** **for all** $W_{i}^{\left(0\right)}=$ *NA* **do** Metropolis–Hastings draw using a random normal walk centered at $W_{i}^{\left(m-1\right)}$
**end if**
**end if**
**if** $W\in R^{a}$ with a>1 **then**
repeat **a**, **b**, and **c** for every W
**end if**
**end for**
**Repeat** steps **1**, **2**, **3**, and **4** to obtain K complete datasets

0. The algorithm is initialized with values for $\alpha_{0}$, α, and β obtained by applying a Cox PH cure model to the complete‐case dataset. By using a Breslow estimator (Breslow 1974) on the same data, we obtain the initial values for $H_{0}\left(t\right)$. The missing cases of W are randomly filled in.
1. The first step consists in estimating $H_{0}\left(Y\right)$, the baseline cumulative hazard rate for the individual times $\left(Y_{1},\text{…},Y_{n}\right)$ using the estimator in (5).
2. In the second step, we compute the estimates of the model parameters $\alpha_{0}$, α, and β. For $\alpha_{0}$ and α, this is done by fitting a logistic model with outcome G and covariates X. Initially, the cure status G has missing values for those censored units not in the right tail of the survival distribution (see step 3). In subsequent iterations, G is filled in with the imputations obtained at the previous iteration. We then draw values $\left(\alpha_{0}^{*},\alpha^{*}\right)$ from a multivariate Gaussian distribution, by using the asymptotic distribution of the ML estimates, for example, MVN $\left(\left({\hat{\alpha }}_{0},\hat{\alpha }\right),{\hat{\Sigma }}_{\left(\alpha_{0},\alpha \right)}\right)$. For β, this is done by fitting a Cox model to the observations where G=1, with follow‐up times Y, event indicator Δ, and covariates Z. We draw values $\beta^{*}$ from MVN $\left(\hat{\beta },{\hat{\Sigma }}_{\beta }\right)$.
3. The following step consists of imputing the cure status G, which is known to be 1 for uncensored observations and assumed to be 0 for censored individuals with follow‐up times after a certain cutoff point. As suggested in the literature (Sy and Taylor 2000), we take the cutoff point equal to the last uncensored follow‐up time. For censored observations until the cutoff point, G is assumed to be MAR conditional on Y and Δ (Beesley et al. 2016), and it can be imputed using its exact conditional distribution
$$\begin{array}{cc} & \mathrm{logit}\left[P(G_{i}=1|X_{i},Z_{i},Y_{i},\Delta_{i}=0)\right]\\ & \;=\log\left[\frac{S_{u}\left(Y_{i}|Z_{i}\right)\pi \left(X_{i}\right)}{1-\pi (X_{i})}\right]\\ & \;=-H_{0}\left(Y_{i}\right)e^{\beta^{T}Z_{i}}+\left(\alpha_{0}+\alpha^{T}X_{i}\right), \end{array}$$
  which is derived from the likelihood introduced in (4). For the sake of clarity, let us recall that both Z and X include the variable with missing cases W.
4. Finally, we impute each missing covariate sequentially using the conditional distributions given in (8) or (9).

Each step makes use of the most recently imputed values of the incomplete variables and the most recently estimated model parameters.

### 3.3 Approximation of the Conditional Distribution

Drawing values from the exact conditional distributions in (8) and (9) can be computationally intensive. Indeed, M–H algorithms are slowed down by the computation of complex target distributions and due to the slow convergence of such algorithms, one may need a very large number of iterations. Beesley et al. (2016) proposed an approximation and compared it to three alternatives from the literature. A simulation study indicated that the proposed approximation outperforms the others in terms of bias when the data have a cure fraction and X=Z. We extend this approach to cases where the sets of incidence and latency covariates differ. As in Beesley et al. (2016), we make use of the first‐order expansion

(10) $$\begin{array}{c} \log\left(1+z\right)\approx \log\left(1+c\right)+\frac{z-c}{1+c} \end{array}$$

if z is near c, and

(11) $$\begin{array}{c} e^{aX+bY}\approx e^{a\bar{X}+b\bar{Y}}\left[1+a\left(X-\bar{X}\right)+b\left(Y-\bar{Y}\right)\right] \end{array}$$

if Var(aX+bY) is small. The only constraint to consider for the approximation (10) to hold and be valid is that the argument of the logarithm function, 1+z and 1+c, must be positive. Therefore, z>−1 and c>−1.

First, we start discussing the case when $W|X^{\left(-j\right)},Z^{\left(-l\right)}∼N\left(\mu ,\sigma \right)$. The log of the exact conditional distribution of W is given by

$$\begin{array}{cc} & \log\left(f(W_{i}|X_{i}^{\left(-j\right)},Z_{i}^{\left(-l\right)},Y_{i},\Delta_{i},G_{i})\right)\\ & \;=G_{i}\Delta_{i}\beta_{l}W_{i}-G_{i}H_{0}\left(Y_{i}\right)e^{\beta^{T}Z_{i}}+G_{i}\alpha_{j}W_{i}+\\ & -\log\left(1+e^{\alpha_{0}+\alpha^{T}X_{i}}\right)-\frac{1}{2\sigma^{2}}{\left(W_{i}-\mu_{i}\right)}^{2}+C, \end{array}$$

where C is a general additive term constant on $W_{i}$. Applying (10), (11), and a second‐order Taylor approximation, one obtains

$$\begin{array}{cc} & \log\left(f(W_{i}|X_{i}^{\left(-j\right)},Z_{i}^{\left(-l\right)},Y_{i},\Delta_{i},G_{i})\right)\\ & \;\approx -\frac{1}{2\sigma^{2}}W_{i}^{2}+\left[G_{i}\Delta_{i}\beta_{l}-G_{i}H_{0}\left(Y_{i}\right)e^{\beta^{T}\bar{Z}}\beta_{l}\right]W_{i}\tilde{C}+\\ & \;+\left[G_{i}\alpha_{j}-\frac{e^{\alpha_{0}+\alpha^{T}\bar{X}}}{1+e^{\alpha_{0}+\alpha^{T}\bar{X}}}\alpha_{j}+\frac{1}{\sigma^{2}}\mu_{i}\right]W_{i}\tilde{C}, \end{array}$$

where $\tilde{C}$ is another constant with respect to $W_{i}$. Recalling that $\mu_{i}$ is a linear combination of $X_{i}^{\left(-j\right)}$ and $Z_{i}^{\left(-l\right)}$, one can see that the distribution of $W_{i}|X_{i}^{\left(-j\right)},Z_{i}^{\left(-l\right)},Y_{i},\Delta_{i},G_{i}$ is approximately Normal, where the mean is a linear combination of $X_{i}^{\left(-j\right)}$, $Z_{i}^{\left(-l\right)}$, $G_{i}$, $G_{i}\Delta_{i}$, and $G_{i}H_{0}\left(Y_{i}\right)$. Then, instead of using the known expressions for the coefficients of this linear approximation, we fit a linear regression model with these covariates to approximate the exact distribution of W.

Second, if we assume that $W|X^{\left(-j\right)},Z^{\left(-l\right)}∼Ber\left(\mathrm{expit}\left(\mu \right)\right)$, the conditional distribution o

$W_{i}|X_{i}^{\left(-j\right)},Z_{i}^{\left(-l\right)},Y_{i},\Delta_{i},G_{i}$ can be approximated using (10) and (11), obtaining

$$\begin{array}{cc} & \mathrm{logit}\left[P(W_{i}=1|X_{i}^{\left(-j\right)},Z_{i}^{\left(-l\right)},Y_{i},\Delta_{i},G_{i})\right]\\ & \;\approx G_{i}\Delta_{i}\beta_{l}-G_{i}H_{0}\left(Y_{i}\right)\left(e^{\beta_{l}}-1\right)e^{\sum_{s\neq l}^{d}\beta_{s}{\bar{Z}}_{s}}\left[1+\sum_{s\neq l}^{d}\beta_{s}\left(Z_{i,s}-{\bar{Z}}_{s}\right)\right]\\ & \;\;+G_{i}\alpha_{j}+\frac{e^{\alpha_{0}+\sum_{b\neq j}^{p}\alpha_{b}{\bar{X}}_{b}}}{1+e^{\alpha_{0}+\sum_{b\neq j}^{p}\alpha_{b}{\bar{X}}_{b}}}\left[1+\sum_{b\neq j}^{p}\alpha_{b}\left(X_{i,b}-{\bar{X}}_{b}\right)\right]\\ & \;\;-\frac{e^{\alpha_{0}+\alpha_{p}+\sum_{b\neq j}^{p}\alpha_{b}{\bar{X}}_{b}}}{1+e^{\alpha_{0}+\alpha_{p}+\sum_{b\neq j}^{p}\alpha_{b}{\bar{X}}_{b}}}\left[1+\sum_{b\neq j}^{p}\alpha_{b}\left(X_{i,b}-{\bar{X}}_{b}\right)\right]+\mu_{i}+C. \end{array}$$

This is a linear combination of $X_{i}^{\left(-j\right)}$, $Z_{i}^{\left(-l\right)}$, $G_{i}$, $G_{i}\Delta_{i}$, $G_{i}H_{0}\left(Y_{i}\right)$, and $G_{i}H_{0}\left(Y_{i}\right)Z_{i}^{\left(-l\right)}$. Therefore, the exact distribution of W can be approximated by fitting a logistic regression model with these covariates as the last step of the imputation procedure presented in Section 3.2.

Details about the scenarios where the variable with missing values W belongs only to the incidence or the latency model are provided in Appendix B. If W belongs only to the incidence component, the conditional distribution of W can be approximated by linear or logistic regression with covariates $X_{i}^{\left(-j\right)}$, $Z_{i}^{\left(-l\right)}$, and $G_{i}$. When W belongs only to the latency, the conditional distribution of W can be approximated by linear or logistic regression with covariates $X_{i}^{\left(-j\right)}$, $Z_{i}^{\left(-l\right)}$, $G_{i}\Delta_{i}$, $G_{i}H_{0}\left(Y_{i}\right)$, and (for *Case 2*) $G_{i}H_{0}\left(Y_{i}\right)Z_{i}^{\left(-l\right)}$.

## 4 Simulation

### 4.1 Simulation Design

We conducted a simulation study to compare the performance of the imputation methods for cure models presented in Section 3. The comparison was carried out considering several scenarios, defined by varying the missing data percentages, model parameters, and variables' distributions.

In this simulation study, we generated 1000 datasets, each containing 500 observations. The variables used in the study are W, X, and Z. Both X and Z follow a Bernoulli distribution with a probability of 0.5, while W is either Ber(0.5) or N(0.5,1). The choice of a Normal variable with a mean of 0.5 rather than a standard Normal was made to ensure the stability of the cure rate when varying W’s distribution. The three variables have a correlation coefficient of 0.5.

Variable X is a covariate in the incidence model, Z is a covariate in the latency model, and both have no missing values. Variable W is a covariate in the models for both incidence and latency, and it is either MCAR with 15% or 30% missing values or MAR with 30% missing observations. To generate MAR data, we considered two distinct mechanisms. In the first mechanism, the missingness of W depends on X,Z, and the outcome (Y,Δ). We used a multivariate amputation procedure (Schouten et al. 2018) implemented in the ampute function from the mice package, setting the missingness proportion to 30% and specifying the pattern (1,1,0,1,1,1), so that only W could be made incomplete. The probability of W being missing was computed through the logistic transformation of the weighted sum of the fully observed variables (X,Z,Y,Δ), using the default MAR weights (positive equal weights for observed variables, zero for W). In the second mechanism, the missingness of W depends only on X and Z, but not on the outcome. This scenario was generated using the same amputation pattern and missingness proportion, but assigning positive MAR weights exclusively to X and Z.

The time to event T is generated from a Weibull PH model of the form

$$\begin{array}{c} S\left(t\right)=\exp\left(-\lambda t^{\rho }\exp\left(\beta_{W}W+\beta_{Z}Z\right)\right) \end{array}$$

with λ=0.25, ρ=1.45, truncated at t=8, to satisfy the sufficient follow‐up assumption. For cured individuals (having G=0), T is set to an extremely large value, as they never experience the event of interest.

The time to censoring C is drawn from an exponential distribution with a parameter equal to 0.08 when W is Bernoulli distributed, and to 0.1 when W is Normally distributed. Both censoring distributions are truncated at 10.

The complete uncured indicator G is generated as a Bernoulli random variable with probability π defined by a logistic regression

(12) $$\begin{array}{c} P\left(G=1\right)=\frac{\exp\left(\alpha_{0}+\alpha_{W}W+\alpha_{X}X\right)}{1+\exp\left(\alpha_{0}+\alpha_{W}W+\alpha_{X}X\right)}. \end{array}$$

In practice, we only use the complete indicator drawn from (12) for generating the follow‐up times Y, namely, to assign large values of Y to cured individuals. Instead, when performing multiple imputation in the cure model, we use the observed uncure indicator, which is 1 for uncensored observations, 0 for observations in the plateau, and unknown otherwise.

We consider two sets of parameter values for the incidence and latency models. The first one is $\alpha_{0}=1$, $\alpha_{W}=-1$, $\alpha_{X}=0.5$, $\beta_{W}=-0.2$, and $\beta_{Z}=0$. This set of parameters is based on the motivating application and therefore is only used when W is Bernoulli distributed with around 15% of observations MCAR. This scenario leads to an average cure rate of ∼33%, an average censoring rate of ∼45%, and an average fraction of observations in the plateau of ∼18%.

The second set of parameters is $\alpha_{0}=0.1$ and $\alpha_{W}=\alpha_{X}=\beta_{W}=\beta_{Z}=0.5$. These values are chosen to illustrate a more general setting. For this choice of parameters, we consider W as Normally distributed with around 30% of observations MAR or MCAR. Both the scenarios in which the missing mechanism is MCAR and MAR lead to an average cure rate of ∼36%, an average censoring rate of ∼46%, and an average fraction of observations in the plateau of ∼17%.

To summarize this simulation design, the four primary scenarios are the following:

i. **Scenario A:** MCAR data with 15% missingness;
W∼ Bernoulli; $\alpha_{0}=1$, $\alpha_{W}=-1$, $\alpha_{X}=0.5$, $\beta_{W}=-0.2$, and $\beta_{Z}=0$.
ii. **Scenario B:** MCAR data with 30% missingness;
W∼ Normal; $\alpha_{0}=0.1$ and $\alpha_{W}=\alpha_{X}=\beta_{W}=\beta_{Z}=0.5$.
iii. **Scenario C:** MAR data with 30% missingness depending on X,Z, and (Y,Δ);
W∼ Normal; $\alpha_{0}=0.1$ and $\alpha_{W}=\alpha_{X}=\beta_{W}=\beta_{Z}=0.5$.
iv. **Scenario D:** MAR data with 30% missingness depending only on X and Z;
W∼ Normal; $\alpha_{0}=0.1$ and $\alpha_{W}=\alpha_{X}=\beta_{W}=\beta_{Z}=0.5$.

#### 4.1.1 Comparison With a Full Model

In addition to these primary scenarios, we also consider Scenario E, corresponding to the method proposed by Beesley et al. (2016), which we will refer to as *full model* and serves as a benchmark for evaluating a further feature of the approach we propose here. In this scenario, we include in both components covariates whose impact on the outcome is null ($\alpha_{Z}=\beta_{X}=0$). In other words, we saturate the model by including irrelevant covariates in the incidence and the latency.

Thus, the set of parameters can be summarized as follows:

v. **Scenario E:** MAR data with 30% missingness depending on X,Z, and (Y,Δ);
W∼ Normal; $\alpha_{0}=0.1$, $\alpha_{W}=\alpha_{X}=\beta_{W}=\beta_{Z}=0.5$, and $\alpha_{Z}=\beta_{X}=0$

For all scenarios, the multiple imputation procedure using the exact conditional distribution in the case of Gaussian W was performed using a Normal distribution with unit variance in the M–H sampling algorithm. The sampler runs for 500 iterations before recording results with 100 iterations between successive samples. For the multiple imputation procedure using the approximate conditional distributions, we use the mice R package (van Buuren and Groothuis‐Oudshoorn 2011).

Following the recommendations of White et al. (2011), we set the number of imputed datasets (in each algorithm iteration) to 15 when the proportion of missing data is 15% (Scenario A) and to 30 for all scenarios with 30% missingness. The number of chained‐equation iterations is set to 10 (Pan and Wei 2016), a choice supported by additional sensitivity checks performed with 20 iterations, which showed negligible differences.

There are two packages to estimate cure models in R, smcure (Cai et al. 2012) and curephEM (Hou et al. 2018). We used the latter with the default values for the convergence criteria and bootstrap sampling for standard error estimation.

### 4.2 Simulation Results

In Tables 1 and 2, we present the results of the simulations for the five different scenarios (A–E).

**TABLE 1:** Simulation study results. Evaluation metrics, B=1000 samples.

Mean absolute bias (MSE) MCSE							
Method	$\alpha_{0}$	$\alpha_{W}$	$\alpha_{X}$	$\alpha_{Z}$	$\beta_{W}$	$\beta_{X}$	$\beta_{Z}$
**Scenario A**	$\alpha_{0}=1$	$\alpha_{W}=-1$	$\alpha_{X}=0.5$		$\beta_{W}=-0.2$		$\beta_{Z}=0$
**(15% MCAR)**							
*Full data*	0.15 (0.04) 0.01	0.21 (0.07) 0.01	0.21 (0.07) 0.01		0.11 (0.02) 0.00		0.11 (0.02) 0.00
*Complete‐case*	0.17 (0.04) 0.01	0.23 (0.08) 0.01	0.23 (0.08) 0.01		0.12 (0.02) 0.00		0.12 (0.02) 0.00
*Exact*	0.15 (0.04) 0.01	0.22 (0.08) 0.01	0.21 (0.07) 0.01		0.12 (0.02) 0.00		0.11 (0.02) 0.00
*Approximate*	0.16 (0.04) 0.01	0.23 (0.08) 0.01	0.21 (0.07) 0.01		0.12 (0.02) 0.00		0.11 (0.02) 0.00
**Scenario B**	$\alpha_{0}=0.1$	$\alpha_{W}=0.5$	$\alpha_{X}=0.5$		$\beta_{W}=0.5$		$\beta_{Z}=0.5$
**(30% MCAR)**							
*Full data*	0.12 (0.02) 0.00	0.10 (0.02) 0.00	0.18 (0.05) 0.01		0.06 (0.01) 0.00		0.11 (0.02) 0.00
*Complete‐case*	0.14 (0.03) 0.01	0.12 (0.02) 0.00	0.22 (0.07) 0.01		0.07 (0.01) 0.00		0.13 (0.03) 0.01
*Exact*	0.13 (0.03) 0.00	0.12 (0.02) 0.00	0.22 (0.08) 0.01		0.08 (0.01) 0.00		0.14 (0.03) 0.00
*Approximate*	0.13 (0.02) 0.00	0.12 (0.02) 0.00	0.19 (0.06) 0.01		0.07 (0.01) 0.00		0.11 (0.02) 0.00
**Scenario C**	$\alpha_{0}=0.1$	$\alpha_{W}=0.5$	$\alpha_{X}=0.5$		$\beta_{W}=0.5$		$\beta_{Z}=0.5$
**(30% MAR** ^**a**^ **)**							
*Full data*	0.12 (0.02) 0.00	0.11 (0.02) 0.00	0.18 (0.05) 0.01		0.06 (0.01) 0.00		0.11 (0.02) 0.00
*Complete‐case*	0.55 (0.35) 0.01	0.16 (0.04) 0.01	0.29 (0.13) 0.01		0.07 (0.01) 0.00		0.13 (0.03) 0.01
*Exact*	0.13 (0.03) 0.00	0.11 (0.02) 0.00	0.22 (0.07) 0.01		0.08 (0.01) 0.00		0.13 (0.02) 0.00
*Approximate*	0.13 (0.02) 0.00	0.16 (0.04) 0.01	0.20 (0.06) 0.01		0.08 (0.01) 0.00		0.11 (0.02) 0.00
**Scenario D**	$\alpha_{0}=0.1$	$\alpha_{W}=0.5$	$\alpha_{X}=0.5$		$\beta_{W}=0.5$		$\beta_{Z}=0.5$
**(30% MAR** ^**b**^ **)**							
*Full data*	0.12 (0.02) 0.00	0.10 (0.02) 0.00	0.19 (0.06) 0.01		0.06 (0.01) 0.00		0.12 (0.02) 0.00
*Complete‐case*	0.14 (0.03) 0.01	0.13 (0.03) 0.01	0.24 (0.09) 0.01		0.08 (0.01) 0.00		0.15 (0.03) 0.01
*Exact*	0.12 (0.02) 0.00	0.12 (0.02) 0.00	0.25 (0.09) 0.01		0.12 (0.02) 0.00		0.19 (0.05) 0.00
*Approximate*	0.13 (0.03) 0.01	0.14 (0.03) 0.01	0.20 (0.06) 0.01		0.09 (0.01) 0.00		0.13 (0.03) 0.01
**Scenario E**	$\alpha_{0}=0.1$	$\alpha_{W}=0.5$	$\alpha_{X}=0.5$	$\alpha_{Z}=0$	$\beta_{W}=0.5$	$\beta_{X}=0$	$\beta_{Z}=0.5$
**(30% MAR** ^**c**^ **)**							
*Full data*	0.14 (0.03) 0.01	0.11 (0.02) 0.00	0.20 (0.07) 0.01	0.20 (0.06) 0.01	0.07 (0.01) 0.00	0.12 (0.02) 0.00	0.12 (0.02) 0.00
*Complete‐case*	0.53 (0.35) 0.01	0.16 (0.04) 0.01	0.31 (0.16) 0.01	0.30 (0.14) 0.01	0.08 (0.01) 0.00	0.14 (0.03) 0.00	0.14 (0.03) 0.01
*Exact*	0.16 (0.04) 0.01	0.12 (0.02) 0.00	0.22 (0.08) 0.01	0.22 (0.07) 0.01	0.09 (0.01) 0.00	0.13 (0.03) 0.00	0.14 (0.03) 0.00
*Approximate*	0.15 (0.03) 0.01	0.17 (0.05) 0.01	0.21 (0.07) 0.01	0.21 (0.07) 0.01	0.08 (0.01) 0.00	0.12 (0.02) 0.00	0.12 (0.02) 0.00

Abbreviations: MSE, mean squared error; MCSE, Monte Carlo standard error.

^a^ Depending on (X,Z,Y,Δ).

^b^ Depending on (X,Z).

^c^ Saturated model.

**TABLE 2:** Simulation study results. Evaluation metrics, B=1000 samples.

CI width (Coverage)							
Method	$\alpha_{0}$	$\alpha_{W}$	$\alpha_{X}$	$\alpha_{Z}$	$\beta_{W}$	$\beta_{X}$	$\beta_{Z}$
**Scenario A**	$\alpha_{0}=1$	$\alpha_{W}=-1$	$\alpha_{X}=0.5$		$\beta_{W}=-0.2$		$\beta_{Z}=0$
**(15% MCAR)**							
*Full data*	0.74 (0.94)	0.98 (0.93)	0.96 (0.94)		0.56 (0.95)		0.54 (0.95)
*Complete‐case*	0.80 (0.95)	1.07 (0.94)	1.05 (0.94)		0.61 (0.95)		0.59 (0.95)
*Exact*	0.75 (0.95)	1.06 (0.94)	0.97 (0.94)		0.60 (0.95)		0.55 (0.95)
*Approximate*	0.76 (0.95)	1.06 (0.94)	0.98 (0.94)		0.60 (0.95)		0.55 (0.95)
**Scenario B**	$\alpha_{0}=0.1$	$\alpha_{W}=0.5$	$\alpha_{X}=0.5$		$\beta_{W}=0.5$		$\beta_{Z}=0.5$
**(30% MCAR)**							
*Full data*	0.60 (0.95)	0.50 (0.95)	0.93 (0.96)		0.31 (0.97)		0.57 (0.98)
*Complete‐case*	0.72 (0.95)	0.61 (0.96)	1.12 (0.96)		0.37 (0.97)		0.69 (0.95)
*Exact*	0.60 (0.93)	0.58 (0.95)	0.90 (0.90)		0.35 (0.94)		0.57 (0.91)
*Approximate*	0.61 (0.95)	0.64 (0.97)	0.98 (0.96)		0.38 (0.99)		0.61 (0.98)
**Scenario C**	$\alpha_{0}=0.1$	$\alpha_{W}=0.5$	$\alpha_{X}=0.5$		$\beta_{W}=0.5$		$\beta_{Z}=0.5$
**(30% MAR** ^**a**^ **)**							
*Full data*	0.60 (0.95)	0.50 (0.95)	0.93 (0.97)		0.31 (0.95)		0.57 (0.98)
*Complete‐case*	0.83 (0.24)	0.74 (0.94)	1.38 (0.95)		0.36 (0.96)		0.65 (0.97)
*Exact*	0.61 (0.94)	0.62 (0.97)	0.90 (0.92)		0.35 (0.92)		0.57 (0.95)
*Approximate*	0.63 (0.95)	0.83 (0.98)	1.05 (0.97)		0.37 (0.95)		0.60 (0.98)
**Scenario D**	$\alpha_{0}=0.1$	$\alpha_{W}=0.5$	$\alpha_{X}=0.5$		$\beta_{W}=0.5$		$\beta_{Z}=0.5$
**(30% MAR** ^**b**^ **)**							
*Full data*	0.60 (0.94)	0.50 (0.94)	0.93 (0.94)		0.31 (0.94)		0.57 (0.95)
*Complete‐case*	0.65 (0.94)	0.60 (0.94)	1.15 (0.94)		0.39 (0.94)		0.70 (0.94)
*Exact*	0.59 (0.94)	0.58 (0.93)	0.89 (0.86)		0.36 (0.80)		0.56 (0.78)
*Approximate*	0.61 (0.95)	0.67 (0.96)	0.99 (0.95)		0.40 (0.94)		0.62 (0.95)
**Scenario E**	$\alpha_{0}=0.1$	$\alpha_{W}=0.5$	$\alpha_{X}=0.5$	$\alpha_{Z}=0$	$\beta_{W}=0.5$	$\beta_{X}=0$	$\beta_{Z}=0.5$
**(30% MAR** ^**c**^ **)**							
*Full data*	0.68 (0.96)	0.53 (0.95)	0.96 (0.94)	0.97 (0.95)	0.32 (0.94)	0.58 (0.94)	0.59 (0.96)
*Complete‐case*	0.95 (0.42)	0.78 (0.94)	1.44 (0.94)	1.42 (0.94)	0.38 (0.94)	0.68 (0.94)	0.69 (0.95)
*Exact*	0.68 (0.92)	0.64 (0.97)	0.94 (0.92)	0.94 (0.92)	0.36 (0.90)	0.59 (0.94)	0.60 (0.92)
*Approximate*	0.74 (0.96)	0.91 (0.98)	1.06 (0.95)	1.08 (0.96)	0.40 (0.95)	0.62 (0.96)	0.62 (0.96)

Abbreviations: CI, confidence interval

^a^Depending on (X,Z,Y,Δ).

^b^Depending on (X,Z).

^c^Saturated model.

The performance of the imputation methods was assessed using different metrics: the mean absolute bias, the mean squared error (MSE), the 95% CI width, the empirical coverage of 95% CIs (Coverage), and the Monte Carlo Standard Error (MCSE), which quantifies the simulation error associated with estimating these metrics across the replicated datasets (Morris et al. 2019). The full data scenario provides a baseline for comparison, as it describes these metrics for models fitted to the original data, without missing values. Note that the full data setting may itself exhibit nonzero absolute bias, reflecting the finite‐sample properties of the mixture cure model estimator rather than any effect of missing data handling; it therefore serves as a natural reference to isolate the additional distortion introduced by missingness and imputation.

In the scenario with 15% MCAR (Scenario A), the CCA, obtained by discarding units with missing values for W and then fitting a cure model on the complete data only, exhibits slightly higher MSE and wider CIs compared to the full data scenario. As theoretically expected under MCAR, estimates from the CCA remain unbiased, with the increase in MSE entirely due to the reduced sample size. Both the exact and the approximate imputation methods performed similarly to the full data scenario, with only minor differences in CI widths, only for the estimates of the effects associated with W. In particular, we observe that multiple imputation lead to a decreased variance for the estimates of the parameters corresponding to fully observed variables.

When the missingness is increased to 30%, still under an MCAR mechanism (Scenario B), this trend is confirmed: the CCA shows higher MSE and wider CIs, while the imputation methods (via either the exact or approximate procedure) yield results close to the full data scenario. Although there are slight variations, these methods effectively preserve empirical coverage, with a slightly greater CI length when the effects of W are considered.

These results show that the proposed approaches provide reliable parameter estimates even in the presence of a moderate to high proportion of missing data. However, the true strength of our methods becomes evident in the MAR scenario (Scenario C). Here, the proposed imputation methods significantly outperform the CCA according to most evaluation metrics. This demonstrates that when data are MAR, both exact and approximate imputation methods can effectively mitigate the impact of missing data, producing estimates close to those obtained with full data. In contrast, the CCA becomes biased, exhibiting a marked increase in MSE and a decrease in coverage. Specifically, for $\alpha_{0}$, the MSE of the complete‐case approach increased to 0.35 and the coverage decreased to 0.24. In addition, the CI width for $\alpha_{X}$ was considerably wider than that obtained when using the imputation methods. Moreover, the estimate $\beta_{Z}$ shows a slight deterioration in terms of CI widths compared to Scenario A. Indeed, when using CCA, the missing data of W leads to the removal of observations that might be critical for accurately estimating $\alpha_{X}$ and $\beta_{Z}$, especially if W is correlated with X and Z. In other words, by eliminating observations with missing W, the model's ability to capture the true relationships involving X and Z may be reduced.

Under Scenario D, where missingness does not depend on the outcome or event indicator, CCA is less biased, and its performance approaches that of MI methods. At the same time, Exact MI shows a slight increase in bias and MSE for some parameters, reflecting unnecessary correction when missingness is independent of the outcome. Although MI produces slightly narrower confidence intervals, coverage decreases notably, particularly for latency‐related parameters. In contrast, the Approximate MI method performs very well in this scenario, achieving bias and coverage close to the full‐data benchmark. These results suggest that the advantage of MI over CCA is context‐dependent: MI is crucial when missingness depends on the outcome, whereas when missingness is unrelated to the outcome, simpler methods like CCA or Approximate MI can provide reliable and efficient inference.

The comparison between Scenarios C and E highlights the advantages of a reduced model with selected covariates in each of the two parts over the full model discussed by Beesley et al. (2016). Focusing on the *Exact* and the *Approximate* methods, we observe consistent improvements in Scenario C across both the incidence and latency components. For instance, the bias for $\alpha_{W}$ decreases from 0.12 in Scenario E to 0.11 in Scenario C using the exact method, and from 0.17 to 0.16 with the approximate method. This improvement is even more evident for $\alpha_{0}$, where the bias decreases from 0.16 (Exact, Scenario E) to 0.13 (Exact, Scenario C), and from 0.15 to 0.13 for the approximate method. In addition, CI widths for all parameters are narrower in Scenario C. Specifically, the CI width for $\alpha_{W}$ improves from 0.64 (Exact, Scenario E) to 0.62 (Exact, Scenario C), and from 0.91 to 0.83 for the approximate method. A similar pattern is observed for $\beta_{W}$, where CI widths decrease from 0.36 (Exact, Scenario E) to 0.35 (Exact, Scenario C), and from 0.40 to 0.37 for the approximate method. More pronounced differences in terms of CI width and coverage are observable for all remaining parameters.

These results demonstrate the advantage of differentiating between covariates relevant to the incidence and to the latency component, as including irrelevant covariates leads to wider confidence intervals. In addition, we also observe that, if all covariates are used in both submodels when not necessary, the approximate method provides more reliable results compared to the exact approach. Even if the differences are modest in this simulation, since the number of parameters in the two models only differs by two, the benefits would be more substantial in scenarios with additional covariates, where the full model risks inefficiency due to overparameterization.

Indeed, while including auxiliary variables in the imputation model can help recover information and reduce bias (Meng 1994), especially under MAR, doing so indiscriminately in the analysis model—particularly in complex settings such as cure models—can lead to increased standard errors. Ideally, one would need to follow a balanced strategy: on the one hand, enriching the imputation model with relevant auxiliary variables to improve the quality of imputation; on the other hand, applying careful variable selection in the analysis model to avoid inefficiency. However, in practice one usually does not have prior information on the relevance of the covariates for the incidence and latency submodel and might still decide to include all of them in both components. In such case, based on the simulation results, we recommend the use of the approximate method instead of the exact conditional distribution.

### 4.3 Algorithm Performance Under Model Misspecification

Appendices A and B provide the exact and approximate distributions for cases where the variable W is included in only the incidence or the latency of the Cox PH cure model. However, we may lack information about the impact of W on the two components. Therefore, we aim to study the performance of the proposed imputation algorithm when the model is misspecified, for example, when W is present in only one of the two components and we assume it is in both. To do this, we conduct a simulation study, examining two further scenarios:

vi. **Scenario F:** MAR data with 30% missingness depending on X,Z, and (Y,Δ);
W∼ Normal; $\alpha_{0}=0.1$, $\alpha_{W}=\alpha_{X}=\beta_{Z}=0.5$, and $\beta_{W}=0$
vii. **Scenario G:** MAR data with 30% missingness depending on X,Z, and (Y,Δ);
W∼ Normal; $\alpha_{0}=0.1$, $\alpha_{X}=\beta_{W}=\beta_{Z}=0.5$, and $\alpha_{W}=0$

These scenarios are similar to Scenario C introduced in Section 4.1, but they differ in that the coefficient associated with the missing covariate W is set to zero in either the latency (Scenario F, $\beta_{W}=0$) or the incidence component (Scenario G, $\alpha_{W}=0$). This allows us to test the algorithm's ability to handle situations where the missing variable affects only one component of the Cox PH cure model. Note however, that these scenarios are not a comparison with Beesley et al. (2016) since the covariates X and Z are not the same.

Importantly, our focus is testing the algorithm's performance under the MAR scenario. We expect that if the algorithm performs well in these more complex conditions, it will likely do so in scenarios where the missing data process is either less complex or it affects a lower portion of the data.

This simulation study provides insights into the robustness of the mixture cure model across the two distinct scenarios. In the previous setting, we discussed the inclusion of irrelevant variables without missing values in both model components, while here we investigate the effect of including a covariate with missing values also when it does not affect one of the submodels. Ideally, we would like a multiple imputation procedure that is robust to such model misspecification. Then, the model itself, through its fit to the available data, can suggest the appropriate placement of different covariates based on the significance of the coefficient estimates.

In addition to these scenarios, we also consider a further misspecification (Scenario H) where the variable W is generated from an Exponential distribution, while during the imputation step we deliberately assume a Normal conditional model for W. This allows us to assess also the robustness of the algorithm when the conditional distribution of the missing variable is misspecified. Similarly to all other scenarios, Scenario H uses the following parameters:

viii. **Scenario H:** MAR data with 30% missingness depending on X,Z, and (Y,Δ);
W∼ Exponential; $\alpha_{0}=0.1$, $\alpha_{W}=\alpha_{X}=\beta_{W}=\beta_{Z}=0.5$

In Tables 3 and 4, simulation results for scenarios F, G, and H are shown. Analogously to what reported in Tables 1 and 2, the performance of the imputation methods was assessed using the mean absolute bias, the MSE, the CI width, the empirical coverage of 95% CIs, and the MCSE.

**TABLE 3:** Simulation results under model misspecification. Evaluation metrics, B=1000 samples.

Absolute Mean Bias (MSE) MCSE					
Method	$\alpha_{0}$	$\alpha_{W}$	$\alpha_{X}$	$\beta_{W}$	$\beta_{Z}$
**Scenario F**	$\alpha_{0}=0.1$	$\alpha_{W}=0.5$	$\alpha_{X}=0.5$	$\beta_{W}=0$	$\beta_{Z}=0.5$
**(30% MAR** ^**a**^ **)**					
*Full data*	0.12 (0.02) 0.00	0.10 (0.02) 0.00	0.20 (0.06) 0.01	0.06 (0.01) 0.00	0.12 (0.02) 0.00
*Complete‐case*	0.54 (0.34) 0.01	0.16 (0.04) 0.01	0.32 (0.16) 0.01	0.08 (0.01) 0.00	0.14 (0.03) 0.01
*Exact*	0.13 (0.03) 0.00	0.16 (0.04) 0.00	0.26 (0.10) 0.01	0.07 (0.01) 0.00	0.12 (0.02) 0.00
*Approximate*	0.12 (0.02) 0.00	0.15 (0.03) 0.01	0.22 (0.08) 0.01	0.07 (0.01) 0.00	0.12 (0.02) 0.00
**Scenario G**	$\alpha_{0}=0.1$	$\alpha_{W}=0$	$\alpha_{X}=0.5$	$\beta_{W}=0.5$	$\beta_{Z}=0.5$
**(30% MAR** ^**a**^ **)**					
*Full data*	0.12 (0.02) 0.00	0.09 (0.01) 0.00	0.18 (0.05) 0.01	0.07 (0.01) 0.00	0.12 (0.02) 0.00
*Complete‐case*	0.49 (0.29) 0.01	0.13 (0.03) 0.01	0.27 (0.12) 0.01	0.08 (0.01) 0.00	0.14 (0.03) 0.01
*Exact*	0.13 (0.02) 0.00	0.14 (0.03) 0.00	0.18 (0.05) 0.01	0.10 (0.01) 0.00	0.15 (0.03) 0.00
*Approximate*	0.12 (0.02) 0.00	0.15 (0.03) 0.01	0.19 (0.06) 0.01	0.09 (0.01) 0.00	0.12 (0.02) 0.00
**Scenario H**	$\alpha_{0}=0.1$	$\alpha_{W}=0.5$	$\alpha_{X}=0.5$	$\beta_{W}=0.5$	$\beta_{Z}=0.5$
**(30% MAR** ^**b**^ **)**					
*Full data*	0.16 (0.04) 0.01	0.11 (0.02) 0.00	0.19 (0.05) 0.01	0.05 (0.00) 0.00	0.10 (0.02) 0.00
*Complete‐case*	0.58 (0.42) 0.01	0.18 (0.05) 0.01	0.31 (0.16) 0.01	0.05 (0.00) 0.00	0.12 (0.02) 0.00
*Exact*	0.15 (0.03) 0.01	0.10 (0.02) 0.00	0.18 (0.05) 0.01	0.06 (0.01) 0.00	0.10 (0.02) 0.00
*Approximate*	0.18 (0.05) 0.01	0.18 (0.05) 0.01	0.19 (0.06) 0.01	0.06 (0.01) 0.00	0.10 (0.02) 0.00

Abbreviations: MSE, mean squared error; MCSE, Monte Carlo standard error.

^a^ Improper placement of W.

^b^ Misspecified distribution for W.

**TABLE 4:** Simulation results under model misspecification. Evaluation metrics, B=1000 samples.

CI width (Coverage)					
Method	$\alpha_{0}$	$\alpha_{W}$	$\alpha_{X}$	$\beta_{W}$	$\beta_{Z}$
**Scenario F**	$\alpha_{0}=0.1$	$\alpha_{W}=0.5$	$\alpha_{X}=0.5$	$\beta_{W}=0$	$\beta_{Z}=0.5$
**(30% MAR** ^**a**^ **)**					
*Full data*	0.59 (0.95)	0.51 (0.96)	0.94 (0.95)	0.30 (0.95)	0.59 (0.96)
*Complete‐case*	0.81 (0.25)	0.75 (0.95)	1.42 (0.94)	0.35 (0.93)	0.68 (0.95)
*Exact*	0.61 (0.94)	0.61 (0.89)	0.91 (0.85)	0.35 (0.96)	0.58 (0.96)
*Approximate*	0.62 (0.95)	0.84 (0.98)	1.07 (0.96)	0.37 (0.96)	0.61 (0.95)
**Scenario G**	$\alpha_{0}=0.1$	$\alpha_{W}=0$	$\alpha_{X}=0.5$	$\beta_{W}=0.5$	$\beta_{Z}=0.5$
**(30% MAR** ^**a**^ **)**					
*Full data*	0.58 (0.94)	0.45 (0.95)	0.89 (0.95)	0.32 (0.95)	0.60 (0.96)
*Complete‐case*	0.79 (0.30)	0.64 (0.95)	1.24 (0.94)	0.37 (0.93)	0.69 (0.95)
*Exact*	0.59 (0.94)	0.56 (0.90)	0.86 (0.94)	0.37 (0.86)	0.60 (0.91)
*Approximate*	0.60 (0.95)	0.76 (0.96)	1.00 (0.96)	0.39 (0.92)	0.64 (0.97)
**Scenario H**	$\alpha_{0}=0.1$	$\alpha_{W}=0.5$	$\alpha_{X}=0.5$	$\beta_{W}=0.5$	$\beta_{Z}=0.5$
**(30% MAR** ^**b**^ **)**					
*Full data*	0.76 (0.95)	0.56 (0.96)	0.88 (0.95)	0.23 (0.96)	0.50 (0.94)
*Complete‐case*	1.11 (0.47)	0.85 (0.95)	1.36 (0.93)	0.26 (0.95)	0.57 (0.96)
*Exact*	0.81 (0.98)	0.70 (0.99)	0.88 (0.94)	0.27 (0.93)	0.51 (0.94)
*Approximate*	0.97 (0.97)	1.01 (0.99)	0.90 (0.95)	0.29 (0.96)	0.52 (0.95)

Abbreviations: CI, confidence interval

^a^Improper placement of W.

^b^Misspecified distribution for W.

As before, the data without missing observations serve as a benchmark.

Quite similar to results in Tables 1 and 2, the CCA shows a significant increase in MSE for $\alpha_{0}$, reaching 0.34 in Scenario F, with a corresponding coverage of only 0.25. This result reflects the method's vulnerability to bias when missing data is not appropriately addressed. The complete‐case method also displays increased CI widths, especially for $\alpha_{X}$, highlighting the loss of information and precision due to missing data.

The exact method generally maintains low MSE values in all scenarios and good coverage for most parameters. However, its performance sometimes falls short of the approximate method, as seen with $\alpha_{W}$ in Scenario F, where the exact method yields a coverage of 0.89 compared to 0.98 of the approximate method. The higher coverage of the approximate method can be attributed to its increased CI width, which provides a more conservative estimate, thereby encompassing more potential values of the parameter. The exact method's performance again suggests it may be more sensitive to model assumptions, leading to increased bias when these assumptions are not met. Despite this, the results support conclusions about the general robustness of the model. The slightly poorer performance compared to the approximate method could be due to the tighter linkage between the missing data variable W and the linear predictors, resulting in higher sensitivity to model assumptions and less flexibility in handling deviations.

The approximate method shows consistent performance with low MSEs in all scenarios and high coverage, highlighting its adaptability in dealing with model deviations. It achieves slightly better coverage for parameters like $\beta_{W}$ in Scenario G, reaching 0.92 compared to the exact method's 0.86.

In Scenario G, the CCA exhibits a substantial increase in MSE values for $\alpha_{0}$ and poor coverage, confirming its inadequacy in scenarios with high levels of missingness. Conversely, the approximate method maintains reliable coverage and CI widths across parameters due to its adaptability and reduced reliance on strict assumptions.

Comparing Scenario C (correctly specified model) with Scenario H (where W is Exponentially distributed but imputed assuming a Normal model), we observe practically identical results across all metrics. This seems to confirm the robustness of the proposed imputation method to misspecification of the covariate distribution, in line with our earlier remark that the conditional models for $W|X^{\left(-j\right)},Z^{\left(-l\right)}$ should be interpreted as working models approximating the true generative mechanism.

Overall, these results suggest that the approximate method is particularly well‐suited for real‐world applications where knowledge of model structure is limited and data are affected by several sources of error. Its robust performance across Scenarios E and F reinforces the idea that it is a viable alternative to the exact method.

## 5 Case Study

Our method is applied to data from the MRC BO06/EORTC 80931 randomized controlled trial (RCT) for patients with localized resectable high‐grade osteosarcoma. Osteosarcoma is a malignant bone tumor which mostly affects children, adolescents, and young adults. Patients with osteosarcoma cannot be successfully treated with surgery alone, but adjuvant chemotherapy has been proven to significantly increase survival (Eilber et al. 1987; Link et al. 1986). The impact of dose intensities is a topic of continuous discussion in this context (Lancia et al. 2019) and it is also relevant to other cancer types. Several previous studies have identified the presence of long‐term disease‐free survivors among osteosarcoma patients, who are practically considered as “cured.” Indeed, less than 5% of patients experience a relapse after five or more years of follow‐up. This implies that a patient falls into one of two groups once the main treatment has been administered: those who have been healed and those who have not, meaning they will eventually suffer from the progression of their disease.

The BO06 clinical trial includes 497 patients diagnosed between May 1993 and September 2002 (Lewis et al. 2007), and randomly assigned to receive either a conventional two‐drug regimen (Reg‐C), consisting of six 3‐week cycles of doxorubicin (DOX, 75 ${\text{mg/m}}^{2}$) and cisplatin (CDDP, 100 ${\text{mg/m}}^{2}$) or a dose‐intensified (DI regimen (Reg‐DI), consisting of the same doses administered twice a week and supported by G‐CSF (5μg/kg daily).

The aim of the MRC BO06/EORTC 80931 clinical trial was to investigate whether increasing the DI would improve the survival of patients with nonmetastatic limb osteosarcoma. The primary outcome measure is PFS since the end of treatment, as a cure could occur at any time during the treatment.

We excluded from the study the patients who did not receive chemotherapy, had abnormal dosages of one or both agents (more than 1.25× prescribed dose), did not have surgery, died or experienced disease progression during treatment (these last two groups were excluded because the primary outcome is PFS calculated from the end of treatment), resulting in a sample of 429 individuals.

Some of the patients' baseline characteristics are shown in Table 5, more details about the patients and chemotherapy can be found in the primary publication of the trial (Lewis et al. 2007).

**TABLE 5:** Baseline characteristics of patients in the BO06 trial.

Characteristic	N=429
**Allocated treatment, n (%)**	
*Reg‐C*	207 (48%)
*Reg‐DI*	222 (52%)
**Sex, n (%)**	
*Female*	173 (40 %)
*Male*	256 (60%)
**Histological response, n (%)**	
*Poor*	214 (56%)
*Good*	165 (44%)
*Unknown*	50

Histological response reflects the proportion of tumor cells that have undergone necrosis after preoperative chemotherapy. A “good” response is defined as more than 90% tumor necrosis, while a “poor” response corresponds to 10% or more viable tumor remaining. Tumors with substantial chondroblastic differentiation are intrinsically less responsive to chemotherapy, and this was assessed to stratify cases; specimens were centrally reviewed whenever possible.

Our purpose is to perform a sensitivity analysis of the results with respect to the method used in handling missing data. Histological response had ∼12% missing values. We expect that the missing mechanism is MCAR since it is probably related to the reporting of the data and there is no reason to believe that it depends on the covariates or the outcome. This setting is consistent with Scenario A in our simulation study, which was specifically designed based on the observed distribution in the motivating data. Multiple imputation of histological response was performed using both exact and approximate approaches.

We fit a Cox PH cure model to the complete data, selecting variables for the incidence and latency components using a combination of clinical knowledge from previous studies (Musta et al. 2022) and statistical considerations. We included histological response and assigned treatment as predictors in the latency component of the model. Conditional on this choice, we selected sex and histological response for the incidence part of the model, as these variables were strongly associated with the probability of being cured. In contrast, previous analyses of the BO06 trial (Musta et al. 2022) found no clear evidence that assigned treatment was predictive of cure, either in univariate models or after conditioning on histological response, with wide confidence intervals crossing the null. For this reason, and to avoid introducing weakly supported predictors in the incidence model, we did not include treatment in this component. Notably, these variables would also have been selected using the penalized likelihood approach proposed by Masud et al. (2018). Details regarding the test for the sufficient follow‐up assumption are provided in Appendix C.

**FIGURE 2:**
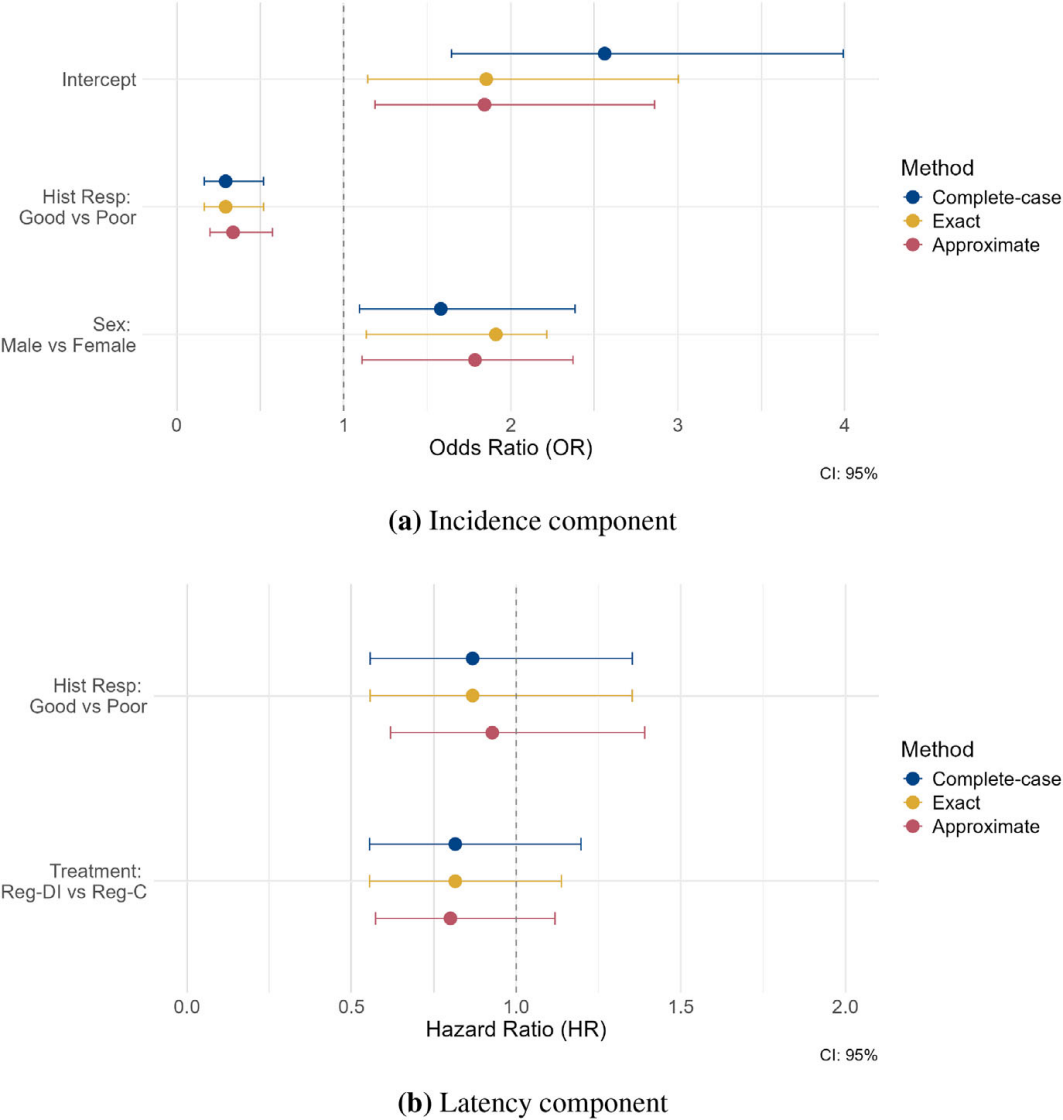
Cox PH cure model for time‐to osteosarcoma recurrence. Odds Ratios for the incidence component and Hazard Ratios for the latency component of the mixture cure models, together with 95% CIs.

Figure 2 illustrates the results of the Cox PH cure model for time‐to‐osteosarcoma recurrence. This figure displays the odds ratios for the incidence component and the hazard ratios for the latency component of the mixture cure models, along with 95% CIs. Panel (a) displays the results for the incidence component, while panel (b) presents those for the latency component.

Point estimates and CIs are very similar between the two imputation approaches. Based on the simulation results, we do not expect big differences between the analyses based on MI or CCA, as the percentage of missingness in the histological response variable is relatively small.

The only minor difference is that CIs tend to be narrower for the imputation approaches than for CCA. The most notable difference between the imputation and complete‐case fits is in the estimates of the model intercept, mainly in the width of his CI, which is much wider in the complete‐case setting. This behavior is consistent with the results observed in the simulations.

As for the simulations, we also compare our extended method with the approach proposed by Beesley et al. (2016), incorporating an additional variable derived from the interaction term of histologic response and treatment.

Table 6 shows the results from the comparison between a full model, which includes all covariates in both the incidence and latency components, and a reduced model, obtained by selecting variables in the two parts.

**TABLE 6:** Comparison of Cox PH cure model estimates with a full and a reduced model for the analysis of osteosarcoma recurrence.

	Full model				Reduced model			
Complete‐case	OR	95% CI	HR	95% CI	OR	95% CI	HR	95% CI
**Intercept**	3.24	1.90, 5.50			2.56	1.65, 3.99		
**Histological response**								
*Poor*	—	—	—	—	—	—	—	—
*Good*	0.19	0.09, 0.42	0.81	0.44, 1.51	0.29	0.17, 0.52	0.87	0.56, 1.35
**Sex**								
*Female*	—	—	—	—	—	—		
*Male*	1.48	1.01, 2.18	1.16	0.87, 1.54	1.58	1.09, 2.28		
**Allocated treatment**								
*Reg‐C*	—	—	—	—			—	—
*Reg‐DI*	0.53	0.25, 1.10	0.90	0.57, 1.42			0.81	0.55, 1.20
Treatment*Hist. resp.								
*Good.Reg‐DI*	2.67	0.92, 7.74	0.99	0.42, 2.31				
**Exact**								
**Intercept**	2.45	1.37, 4.41			1.85	1.14, 3.00		
**Histological response**								
*Poor*	—	—	—	—	—	—	—	—
*Good*	0.19	0.09, 0.42	0.81	0.44, 1.52	0.29	0.17, 0.52	0.87	0.56, 1.35
**Sex**								
*Female*	—	—	—	—	—	—		
*Male*	1.74	1.01, 3.00	1.24	0.84, 1.82	1.91	1.14, 2.22		
**Allocated treatment**								
*Reg‐C*	—	—	—	—			—	—
*Reg‐DI*	0.53	0.25, 1.10	0.90	0.57, 1.42			0.82	0.55, 1.14
Treatment*Hist. resp.								
*Good.Reg‐DI*	2.67	0.92, 7.75	0.99	0.42, 2.31				
**Approximate**								
**Intercept**	2.12	1.24, 3.61			1.84	1.19, 2.86		
**Histological response**								
*Poor*	—	—	—	—	—	—	—	—
*Good*	0.24	0.11, 0.49	0.90	0.50, 1.62	0.34	0.20, 0.57	0.93	0.62, 1.39
**Sex**								
*Female*	—	—	—	—	—	—		
*Male*	1.68	1.01, 2.80	1.18	0.82, 1.70	1.79	1.11, 2.37		
**Allocated treatment**								
*Reg‐C*	—	—	—	—			—	—
*Reg‐DI*	0.75	0.37, 1.53	0.82	0.53, 1.26			0.80	0.57, 1.12
Treatment*Hist. resp.								
*Good.Reg‐DI*	2.12	0.75, 6.03	0.95	0.43, 2.11				

Abbreviations: OR, odds ratio; CI, confidence interval; HR, hazard ratio.

The estimates from the saturated model show a higher uncertainty in parameter estimates. This is evident in the wider CIs, especially for the intercept, sex, and treatment effects, indicating that the inclusion of unnecessary covariates introduces additional variability and reduces the precision of the estimates. In contrast, for the nonsaturated models, both Exact and Approximate, the confidence intervals are narrower. The odds ratios for “Good” histological response are consistent across both imputation methods, but the CIs are tighter in the Exact method, as observed in the simulation study. Overall, we observe that the precision of the parameter estimates improves when we limit the covariates to those that are meaningful in the context of the disease and treatment under study. This suggests that distinguishing between the covariates affecting the probability of being cured and those influencing the survival of uncured patients may be crucial for more efficient modeling.

## 6 Discussion

Our work on imputation methods for mixture cure models in the presence of missing covariate values emphasizes the importance of flexible imputation techniques. In this paper, we investigate the methodology for imputing partially observed covariates in a mixture cure model by accommodating potentially distinct sets of covariates for the cure probability and the survival of uncured patients. This extension is motivated by both theoretical considerations—such as the identifiability benefits of distinct covariate sets—and practical challenges that arise when including all available covariates to both submodels. Importantly, we release the R‐code to implement the proposed approach.

The results show that both exact and approximate imputation approaches produce similar results when the model is correctly specified. In particular, imputation methods yield narrower CIs compared to CCA, enhancing the precision of our estimates. The simulation results suggest that the proposed methods are robust to the presence of missing values, particularly under the MAR scenario, where they outperform the CCA. These findings are consistent with the known properties of CCA: while it remains unbiased under MCAR and can also yield valid results under MAR in certain model settings (Bartlett et al. 2015; Hughes et al. 2019), it generally suffers from reduced efficiency compared to multiple imputation. In settings where CCA is not valid and the MAR assumption holds, multiple imputation substantially improves estimation accuracy and coverage by restoring lost information due to missingness. The exact imputation method slightly outperforms the approximate method in terms of CI width, yielding more precise estimates. However, when the model is misspecified, that is, certain covariates are wrongly included in the incidence or latency components, the approximate method has better coverage probabilities, hence it is a safer and more robust choice for practical applications. Furthermore, the results illustrate the advantage of differentiating covariates between incidence and latency, since wrongly including all covariates in both components leads to wider CIs and worse performance of the exact approach.

For data from the BO06 clinical trial on osteosarcoma, the application of the Cox PH cure model reveals several key findings. First, as observed in the CCA, histological response remains a strong prognostic factor for the cure fraction, indicating that patients who respond well to initial treatment are more likely to be cured. However, even after accounting for missing values, there is no clear indication that a good histological response is associated with longer PFS for uncured patients. This underscores the importance of having the possibility to differentiate between factors that influence the probability of cure from those that affect survival among uncured patients. Overall, we observe that, despite slight changes in the estimated coefficients and in the length of the confidence intervals, the conclusions about the significance of the covariate effects are the same for all the models and estimation procedures considered.

As a further development of this work, one might consider an alternative way of performing multiple imputation based on the observed likelihood (instead of the complete one), which avoids considering the latent cure status as an additional variable with missing values. When there is only one missing variable, this would avoid the chained equations, since there would be no need to impute G. However, such an approach would require the estimation of the parameters α and β in Step 2 of Algorithm 1 by fitting a mixture cure model and the variance needs to be estimated via computationally intensive bootstrap. Hence, we will explore this possibility in future works.

Moreover, while we follow current recommendations by including the cumulative baseline hazard estimate in the imputation model to improve compatibility with the Cox model, as proposed by Beesley et al. (2016), this approach does not ensure full model compatibility. As shown in Bartlett et al. (2015), incompatibility may arise when default imputation models are used for covariates in nonlinear substantive models. In such cases, unless the imputation model is restricted in a way that makes it compatible with the substantive model, misspecification can lead to inconsistent estimates. Substantive model–compatible FCS addresses this issue by embedding the substantive model directly into the imputation procedure. While this approach is already implemented in available software (e.g., the smcfcs package in R (Bartlett et al. 2023)), its application in the context of cure models and complex survival settings may still require further methodological development and adaptation. We plan to investigate this avenue in future work.

We note also that our imputation approach based on the exact conditional distribution is in line with the recommendation of Bartlett et al. (2015) to use an imputation model which is compatible with the substantive model. Indeed, we derived the exact conditional distribution of the missing covariate and of the latent cure status (treated as a variable with missing values), given all the other variables, based on the substantive model and the assumed distribution of the missing covariate given the other covariates. In the continuous case, this resulted in a nonstandard distribution, which is less straightforward to sample from. The approximate method is however no longer compatible, but the simulation results still show good performance, even when the substantive model is misspecified. This is probably because it is still an approximation of the exact distribution of a compatible model.

Finally, variable selection represents an important topic in cure models with missing data. Existing penalized likelihood approaches for cure models (e.g., Liu et al. 2012; Masud et al. 2018) do not explicitly address missingness, and combining them with multiple imputation poses challenging issues related to tuning parameter selection, computational burden, and postselection inference. Possible strategies include performing variable selection within each imputed data set and aggregating the selected sets, as well as stacked or bootstrap‐based procedures proposed in the missing‐data literature (see, e.g., Zhao and Long 2017). A systematic evaluation of such methods, and their adaptation to mixture cure models, constitutes an interesting avenue for future research.

## Conflicts of Interest

The authors declare no conflicts of interest.

## Open Research Badges

This article has earned an Open Data badge for making publicly available the digitally‐shareable data necessary to reproduce the reported results. The data is available in the Supporting Information section.

This article has earned an open data badge “**Reproducible Research**” for making publicly available the code necessary to reproduce the reported results. “The results reported in this article could fully be reproduced.”

## Supporting information


**Supporting File:** bimj70144‐sup‐0001‐DataCode.zip.

## Data Availability

The source codes for implementing the imputation algorithm and for reproducing the simulations are available at https://github.com/martacip/mi_curemodels.

## Endnotes

1 The source code is available at https://github.com/martacip/mi_curemodels.

## References

[bimj70144-bib-0001] Amico, M. , and I. Van Keilegom . 2018. “Cure Models in Survival Analysis.” Annual Review of Statistics and Its Application 5: 311–342.

[bimj70144-bib-0002] Bartlett, J. , O. Harel , and J. Carpenter . 2015. “Asymptotically Unbiased Estimation of Exposure Odds Ratios in Complete Records Logistic Regression.” American Journal of Epidemiology 182, no. 8: 730–736. 26429998 10.1093/aje/kwv114 PMC4597800

[bimj70144-bib-0003] Bartlett, J. , T. Morris , K. White , and I. White . 2023. smcfcs: Substantive Model Compatible Fully Conditional Specification . R Package Version 1.5.5.

[bimj70144-bib-0004] Bartlett, J. , S. Seaman , I. White , and J. Carpenter . 2015. “Multiple Imputation of Covariates by Fully Conditional Specification: Accommodating the Substantive Model.” Statistical Methods in Medical Research 24, no. 4: 462–487. 24525487 10.1177/0962280214521348 PMC4513015

[bimj70144-bib-0005] Beesley, L. J. , J. W. Bartlett , G. T. Wolf , and J. M. Taylor . 2016. “Multiple Imputation of Missing Covariates for the Cox Proportional Hazards Cure Model.” Statistics in Medicine 35, no. 26: 4701–4717. 27439726 10.1002/sim.7048 PMC5053880

[bimj70144-bib-0006] Breslow, N. E. 1974. “Covariates Analysis of Censored Survival Data.” Biometrics 30: 89–99. 4813387

[bimj70144-bib-0007] Cai, C. , Y. Zou , Y. Peng , and J. Zhang . 2012. “smcure: An R‐Package for Estimating Semiparametric Mixture Cure Models.” Computer Methods and Programs in Biomedicine 108, no. 3: 1255–1260. 23017250 10.1016/j.cmpb.2012.08.013 PMC3494798

[bimj70144-bib-0008] Cox, D. 1972. “Regression Models and Life‐Tables.” Journal of the Royal Statistical Society 34, no. 2: 187–202.

[bimj70144-bib-0009] Eilber, F. , A. Giuliano , J. Eckardt , K. Patterson , S. Moseley , and J. Goodnight . 1987. “Adjuvant Chemotherapy for Osteosarcoma: A Randomized Prospective Trial.” Journal of Clinical Oncology 5: 21–26. 3543236 10.1200/JCO.1987.5.1.21

[bimj70144-bib-0010] Farewell, V. T. 1977. “A Model for a Binary Variable With Time‐Censored Observations.” Biometrika 64, no. 1: 43–46.

[bimj70144-bib-0011] Ferrari, S. , A. Briccoli , M. Mercuri , et al. 2006. “Late Relapse in Osteosarcoma.” Journal of Pediatric Hematology/Oncology 28, no. 7: 418–422. 16825986 10.1097/01.mph.0000212944.82361.1d

[bimj70144-bib-0012] Hanin, L. , and L. Huang . 2014. “Identifiability of Cure Models Revisited.” Journal of Multivariate Analysis 130: 261–274.

[bimj70144-bib-0013] Hastings, W. K. 1970. “Monte Carlo Sampling Methods Using Markov Chains and Their Applications.” Biometrika 57, no. 1: 97–109.

[bimj70144-bib-0014] Hou, J. , C. Chambers , and R. Xu . 2018. “A Nonparametric Maximum Likelihood Approach for Survival Data With Observed Cured Subjects, Left Truncation and Right‐Censoring.” Lifetime Data Analysis 24: 612–651. 29238894 10.1007/s10985-017-9415-2

[bimj70144-bib-0015] Hughes, R. , J. Heron , J. Sterne , and K. Tilling . 2019. “Accounting for Missing Data in Statistical Analyses: Multiple Imputation is Not Always the Answer.” International Journal of Epidemiology 48, no. 4: 1294–1304. 30879056 10.1093/ije/dyz032 PMC6693809

[bimj70144-bib-0016] Kuk, A. Y. , and C.‐H. Chen . 1992. “A Mixture Model Combining Logistic Regression With Proportional Hazards Regression.” Biometrika 79, no. 3: 531–541.

[bimj70144-bib-0017] Lancia, C. , J. Anninga , M. Sydes , et al. 2019. “A Novel Method to Address the Association Between Received Dose Intensity and Survival Outcome: Benefits of Approaching Treatment Intensification at a More Individualised Level in a Trial of the European Osteosarcoma Intergroup.” Cancer Chemotherapy and Pharmacology 83, no. 5: 951–962. 30879111 10.1007/s00280-019-03797-3 PMC6458990

[bimj70144-bib-0018] Lee, K. , K. Tilling , R. Cornish , et al. 2021. “Framework for the Treatment and Reporting of Missing Data in Observational Studies: The Treatment and Reporting of Missing Data in Observational Studies Framework.” Journal of Clinical Epidemiology 134: 79–88. 33539930 10.1016/j.jclinepi.2021.01.008 PMC8168830

[bimj70144-bib-0019] Legrand, C. , and A. Bertrand . 2019. “Cure Models in Cancer Clinical Trials.” In Textbook of Clinical Trials in Oncology, 465–492. Chapman and Hall/CRC.

[bimj70144-bib-0020] Lewis, I. , M. Nooij , J. Whelan , et al. 2007. “Improvement in Histologic Response But not Survival in Osteosarcoma Patients Treated With Intensified Chemotherapy: A Randomized Phase III Trial of the European Osteosarcoma Intergroup.” Journal of the National Cancer Institute 99: 112–128. 17227995 10.1093/jnci/djk015

[bimj70144-bib-0021] Link, M. , A. Goorin , A. Miser , et al. 1986. “The Effect of Adjuvant Chemotherapy on Relapse‐Free Survival in Patients With Osteosarcoma of the Extremity.” New England Journal of Medicine 314, no. 25: 1600–1606. 3520317 10.1056/NEJM198606193142502

[bimj70144-bib-0022] Little, R. J. , and D. B. Rubin . 2002. Statistical Analysis With Missing Data. Wiley.

[bimj70144-bib-0023] Liu, X. , Y. Peng , D. Tu , and H. Liang . 2012. “Variable Selection in Semiparametric Cure Models Based on Penalized Likelihood, With Application to Breast Cancer Clinical Trials.” Statistics in Medicine 31, no. 24: 2882–2891. 22733695 10.1002/sim.5378

[bimj70144-bib-0024] Maller, R. , S. Resnick , and S. Shemehsavar . 2024. “Finite Sample and Asymptotic Distributions of a Statistic for Sufficient Follow‐Up in Cure Models.” Canadian Journal of Statistics 52, no. 2: 359–379.

[bimj70144-bib-0025] Masud, A. , W. Tu , and Z. Yu . 2018. “Variable Selection for Mixture and Promotion Time Cure Rate Models.” Statistical Methods in Medical Research 27, no. 7: 2185–2199. 27856963 10.1177/0962280216677748

[bimj70144-bib-0026] Meng, X.‐L. 1994. “Multiple‐Imputation Inferences With Uncongenial Sources of Input.” Statistical Science 9, no. 4: 538–558.

[bimj70144-bib-0027] Metropolis, N. , A. W. Rosenbluth , M. N. Rosenbluth , A. H. Teller , and E. Teller . 1953. “Equation of State Calculations by Fast Computing Machines.” Journal of Chemical Physics 21, no. 6: 1087–1092. 10.1063/5.0309018 41342507

[bimj70144-bib-0028] Morris, T. , I. White , and M. Crowther . 2019. “Using Simulation Studies to Evaluate Statistical Methods.” Statistics in Medicine 38: 2074–2102. 30652356 10.1002/sim.8086 PMC6492164

[bimj70144-bib-0029] Musta, E. , N. van Geloven , J. Anninga , H. Gelderblom , and M. Fiocco . 2022. “Short‐Term and Long‐Term Prognostic Value of Histological Response and Intensified Chemotherapy in Osteosarcoma: A Retrospective Reanalysis of the BO06 Trial.” BMJ Open 12, no. 5: e052941. 10.1136/bmjopen-2021-052941 PMC9092180 35537786

[bimj70144-bib-0030] Pan, Q. , and R. Wei . 2016. “Fraction of Missing Information (γ) at Different Missing Data Fractions in the 2012 NAMCS Physician Workflow Mail Survey.” Applied Mathematics 7, no. 10: 1057–1067. 27398259 10.4236/am.2016.710093 PMC4934387

[bimj70144-bib-0031] Peng, Y. , and K. B. Dear . 2000. “A Nonparametric Mixture Model for Cure Rate Estimation.” Biometrics 56, no. 1: 237–243. 10783801 10.1111/j.0006-341x.2000.00237.x

[bimj70144-bib-0032] Peng, Y. , and B. Yu . 2021. Cure Models: Methods, Applications, and Implementation. Chapman and Hall/CRC.

[bimj70144-bib-0033] Rubin, D. B. 1987. Multiple Imputation for Nonresponse in Surveys. Wiley.

[bimj70144-bib-0034] Rubin, D. B. 2004. Multiple Imputation for Nonresponse in Surveys. Vol. 81. Wiley.

[bimj70144-bib-0035] Schouten, R. , P. Lugtig , and G. Vink . 2018. “Generating Missing Values for Simulation Purposes: A Multivariate Amputation Procedure.” Journal of Statistical Computation and Simulation 88, no. 15: 2909–2930.

[bimj70144-bib-0036] Sy, J. P. , and J. M. Taylor . 2000. “Estimation in a Cox Proportional Hazards Cure Model.” Biometrics 56, no. 1: 227–236. 10783800 10.1111/j.0006-341x.2000.00227.x

[bimj70144-bib-0037] van Buuren, S. 2018. Flexible Imputation of Missing Data. CRC Press.

[bimj70144-bib-0038] van Buuren, S. , J. P. Brand , C. G. Groothuis‐Oudshoorn , and D. B. Rubin . 2006. “Fully Conditional Specification in Multivariate Imputation.” Journal of Statistical Computation and Simulation 76, no. 12: 1049–1064.

[bimj70144-bib-0039] van Buuren, S. , and C. Groothuis‐Oudshoorn . 2011. “mice: Multivariate Imputation by Chained Equations in R.” Journal of Statistical Software 45, no. 3: 1–67.

[bimj70144-bib-0040] White, I. , P. Royston , and A. Wood . 2011. “Multiple Imputation Using Chained Equations: Issues and Guidance for Practice.” Statistics in Medicine 30, no. 4: 377–399. 21225900 10.1002/sim.4067

[bimj70144-bib-0041] White, I. R. , and P. Royston . 2009. “Imputing Missing Covariate Values for the Cox Model.” Statistics in Medicine 28, no. 15: 1982–1998. 19452569 10.1002/sim.3618 PMC2998703

[bimj70144-bib-0042] Xie, P. , M. Escobar‐Bach , and I. van Keilegom . 2023. “Testing for Sufficient Follow‐Up in Censored Survival Data by Using Extremes.” arXiv, Preprint, arXiv:2309.00868 . 10.1002/bimj.202400033 39377280

[bimj70144-bib-0043] Yuen, T. , and E. Musta . 2024. “Testing for Sufficient Follow‐Up in Survival Data With a Cure Fraction.” arXiv, Preprint, arXiv:2403.16832 . 10.1002/bimj.70121 PMC12961184 41782254

[bimj70144-bib-0044] Zhao, Y. , and Q. Long . 2017. “Variable Selection in the Presence of Missing Data: Imputation‐Based Methods.” Wiley Interdisciplinary Reviews: Computational Statistics 9, no. 5: e1402. 29085552 10.1002/wics.1402 PMC5659333

