# Supplementary figures and images for: A Multiple Imputation Approach to Distinguish Curative From Life‐Prolonging Effects in the Presence of Missing Covariates
Source: Biom J. 2026 Jun 8;68(3):e70144. doi: 10.1002/bimj.70144 (PMC13244124; doi:10.1002/bimj.70144)

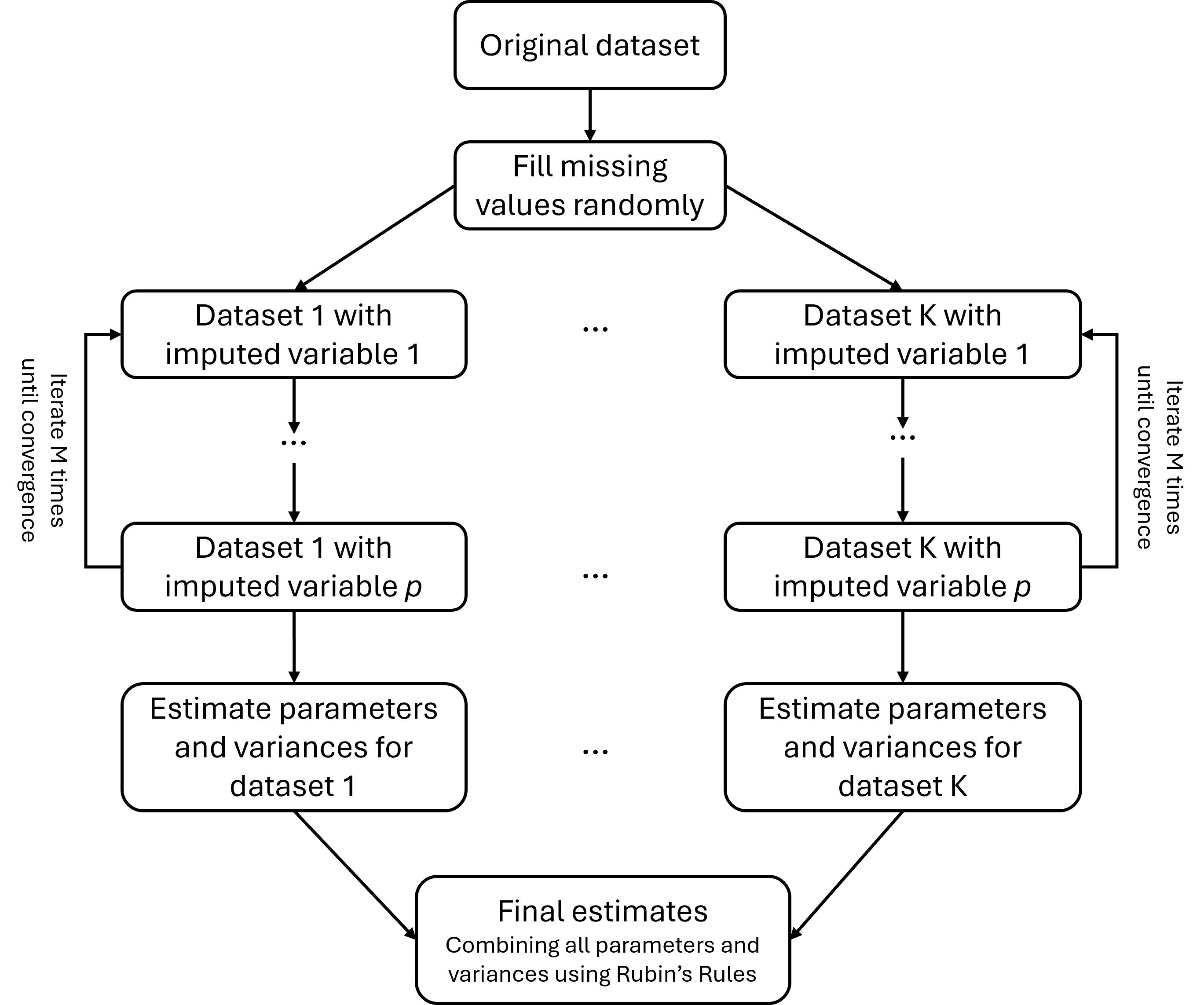

Supplement: Supplementary file 1 — Supporting File: bimj70144‐sup‐0001‐DataCode.zip. [file BIMJ-68-e70144-s001.zip › bimj.202500197_code_data/Code_and_Data/results/Figure1.png]

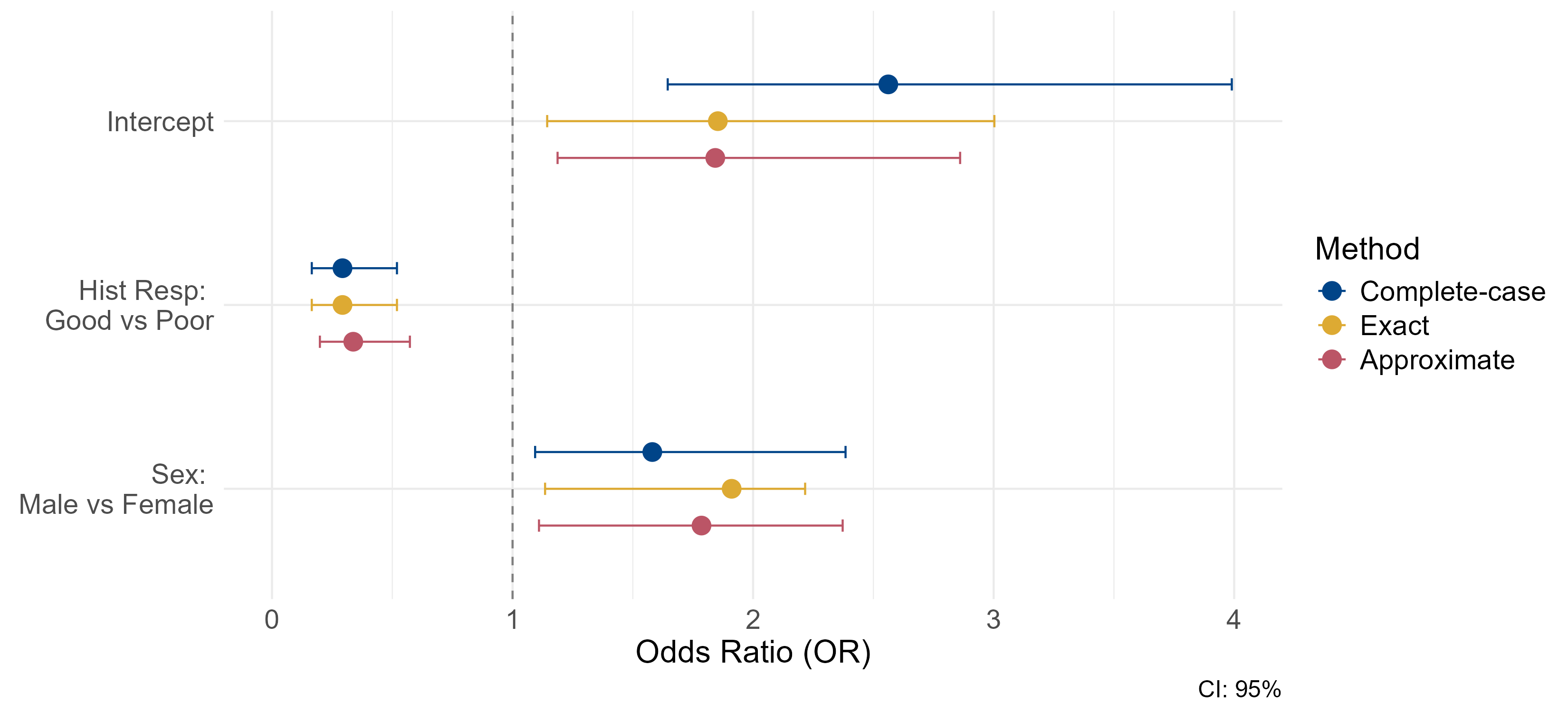

Supplement: Supplementary file 1 — Supporting File: bimj70144‐sup‐0001‐DataCode.zip. [file BIMJ-68-e70144-s001.zip › bimj.202500197_code_data/Code_and_Data/results/Figure2_A.png]

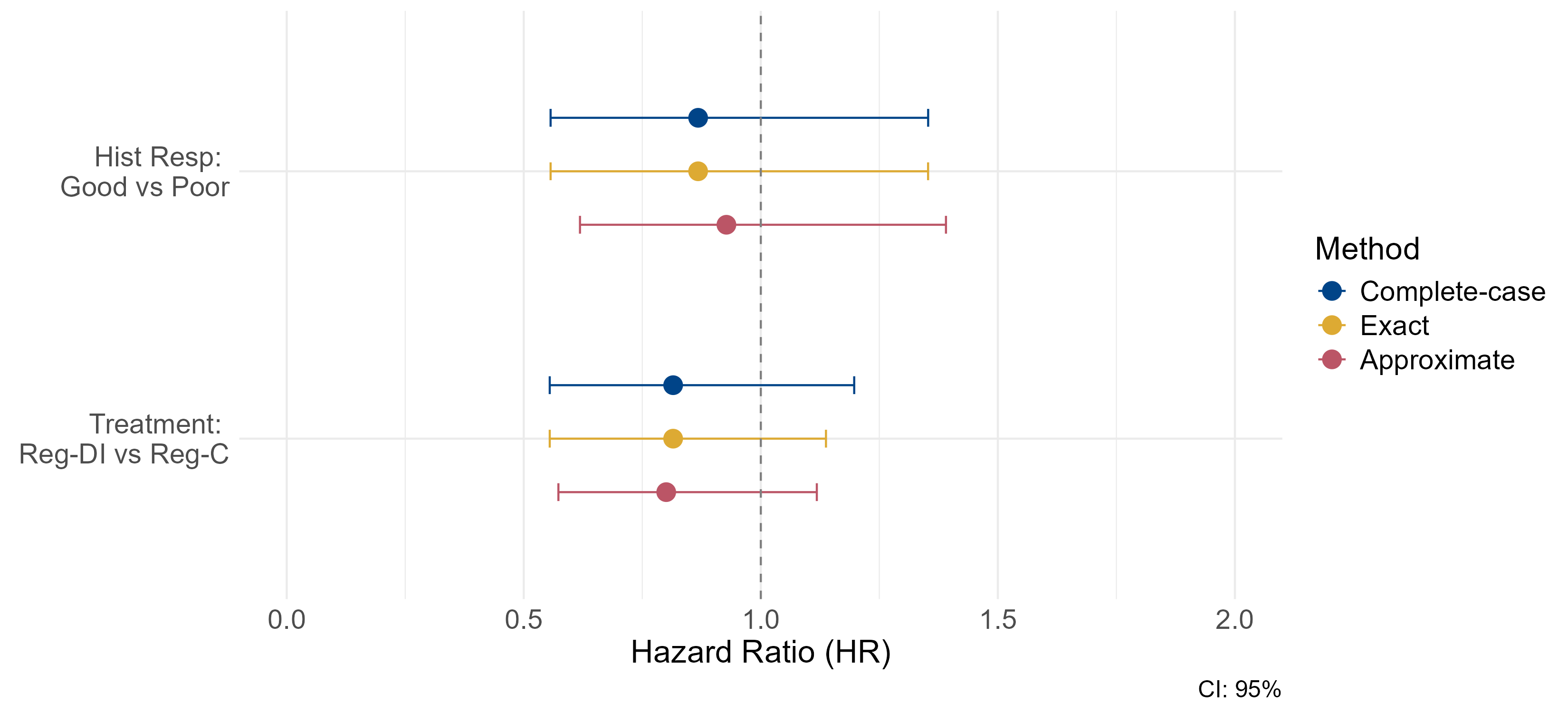

Supplement: Supplementary file 1 — Supporting File: bimj70144‐sup‐0001‐DataCode.zip. [file BIMJ-68-e70144-s001.zip › bimj.202500197_code_data/Code_and_Data/results/Figure2_B.png]
